# Enabling long-lived organic room temperature phosphorescence in polymers by subunit interlocking

**DOI:** 10.1038/s41467-019-11749-x

**Published:** 2019-09-18

**Authors:** Suzhi Cai, Huili Ma, Huifang Shi, He Wang, Xuan Wang, Leixin Xiao, Wenpeng Ye, Kaiwei Huang, Xudong Cao, Nan Gan, Chaoqun Ma, Mingxing Gu, Lulu Song, Hai Xu, Youtian Tao, Chunfeng Zhang, Wei Yao, Zhongfu An, Wei Huang

**Affiliations:** 1 Key Laboratory of Flexible Electronics & Institute of Advanced Materials, Nanjing Tech University, 30 South Puzhu Road, Nanjing, 211816 China; 2 National Laboratory of Solid State Microstructures, School of Physics, Collaborative Innovation Center for Advanced Microstructures, Nanjing University, Nanjing, 210093 China; 3 Institute of Flexible Electronics (IFE), Northwestern Polytechnical University (NPU), 127 West Youyi Road, Xi’an, 710072 China; 4 Key Laboratory for Organic Electronics and Information Displays & Institute of Advanced Materials, Jiangsu National Synergistic Innovation Center for Advanced Materials, Nanjing University of Posts and Telecommunications, Nanjing, 210023 China

**Keywords:** Polymers, Self-assembly, Polymers

## Abstract

Long-lived room temperature phosphorescence (LRTP) is an attractive optical phenomenon in organic electronics and photonics. Despite the rapid advance, it is still a formidable challenge to explore a universal approach to obtain LRTP in amorphous polymers. Based on the traditional polyethylene derivatives, we herein present a facile and concise chemical strategy to achieve ultralong phosphorescence in polymers by ionic bonding cross-linking. Impressively, a record LRTP lifetime of up to 2.1 s in amorphous polymers under ambient conditions is set up. Moreover, multicolor long-lived phosphorescent emission can be procured by tuning the excitation wavelength in single-component polymer materials. These results outline a fundamental principle for the construction of polymer materials with LRTP, endowing traditional polymers with fresh features for potential applications.

## Abstract (web summary)

Long-lived room temperature phosphorescence (LRTP) is important in organic photonics but exploring a universal approach to obtain LRTP in amorphous polymers is challenging. Here the authors present a facile chemical strategy to achieve ultralong phosphorescence in polymers by ionic bonding cross-linking.

## Introduction

**Fig. 1:**
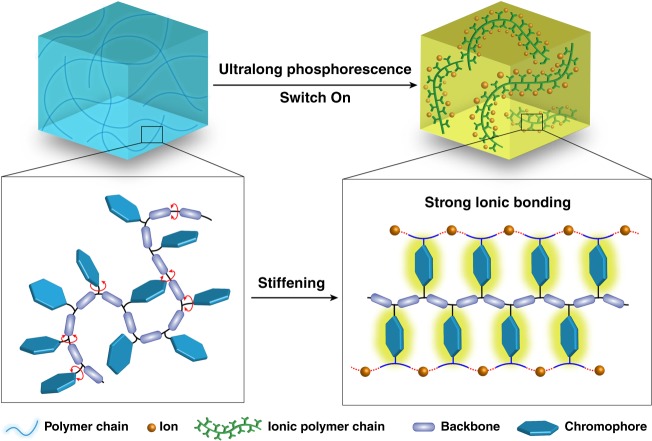
Schematic illustration for ultralong room temperature phosphorescence in polymers. After chromophores interlocked by ions, the typical polymers were endowed with LRTP properties through restricting motions of chromophores under ambient conditions

Persistent luminescence is an attracting optical phenomenon that can last for seconds, minutes, even hours after the cease of irradiation^1,2^, which has aroused extensive attention due to its broad applications ranging from displays and optical storage to sensors and bioimaging^1,3–6^. Recently, a clear trend is emerging, shifting the emphasis from inorganic counterparts to organic luminescent materials with ultralong phosphorescence^5,7,8^, due to their superiorities, such as electroconductivity, diverse molecular architechtures, good biocompatibilities, and low cost, although there still exists a great challenge of suppressing non-radiative transition and enhancing intersystem crossing for organic phosphorescence under ambient conditions. A set of feasible approaches, such as crystallization engineering^8,9^, doping in rigid matrixes^10–13^, H-aggregation^5^, the construction of metal organic frameworks^14^, the formation of carbon dots^15^ and so forth^15–26^, were proposed to obtain ultralong phosphorescence at room temperature. However, these phosphors were mainly limited to crystalline system or doped composites, which greatly hindered their practical applications due to the stringent requirement of the formation of crystalline state and poor processability for small organic compounds, or inevitable phase separation in host-guest systems^10,27^. Considering the intriguing features of polymers in the field of flexible electronics^28–31^, such as flexible, lightweight, good processability, and stretchability, it is necessary to investigate long-lived phosphorescence based on polymers under ambient conditions (in air and at room temperature), which have great potential in flexible lighting and displays. Much effort has been devoted to develop polymers with long-lived phosphorescence, there is still an overwhelming barrier to provide a concise strategy to rationally manipulate polymers with long-lived phosphorescence under ambient conditions because of the intense non-radiative transition of triplet excitons from building block motions (Fig. 1).

Several successful examples for polymers with LRTP mainly depended on hydrogen bonding to restrict the motions of the chromophores^26,27^. Compared with the hydrogen bond, ionic bonding possesses the unique characteristics of strong interactions, directionless, nonsaturation^32,33^. Generally, ionic cross-linking are widely used for bridging building units to obtain superior physical properties in supramolecular chemistry. For instance, the ionic bonding was chosen to construct hydrogels or self-healing materials due to its strong ionic bonding interactions to maintain the shape of the gels and recover original shape from the stretched state^34^. Moreover, for supramolecular polymers, building blocks can be connected through electrostatic interactions of ionic bonds to create a coiled chain or ordered filament^35,36^. Inspired by the strong cross-linking interactions from ionic bonding in material science, we propose that the introduction of ionic bonding in polymers may obtain LRTP via suppressing non-radiative transition process from the motions of chromophores. As shown in Fig. 1, there exists intensive triplet exciton depletion in traditional polymers under ambient conditions on account of the motions of chromophores, thus resulting in no phosphorescence emission. After cross-linked by the ionic bonding, the chromophores can be fixed with covalent bonding of vinyl backbone, effectively stabilizing triplet excitons for LRTP in polymers through the suppression of non-radiative transitions. The LRTP lifetime can reach 2.1 s. Unexpectedly, excitation dependent colorful long-lived phosphorescence is obtained. Our approach provides a general design principle to generate long-lived phosphorescence in amorphous polymers under ambient conditions.

## Results

### Photophysical properties of the PSSNa polymer

**Fig. 2:**
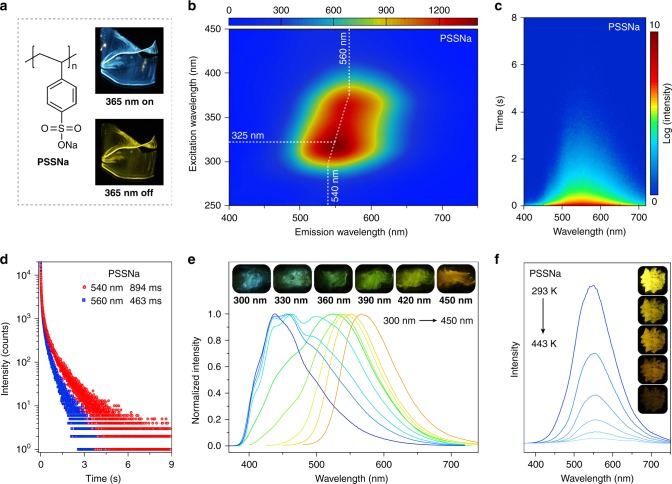
Photophysical properties of dry PSSNa polymer in solid state. **a** Molecular structure of PSSNa polymer. Right: Photographs of PSSNa solids taken under a 365 nm lamp on and off. **b** Excitation-phosphorescence emission mapping of PSSNa polymer under ambient conditions. **c** Time-resolved emission spectra of PSSNa polymer excited by 325 nm. **d** The lifetime profiles of PSSNa polymer monitoring at 540 nm excited by 300 nm and 560 nm excited by 365 nm under ambient conditions, respectively. **e** Excitation dependent phosphorescence spectra of PSSNa polymer at 77 K. Insets show photographs taken after PSSNa polymer excited by 300, 330, 360, 390, 420, and 450 nm at 77 K. **f** Phosphorescence spectra of PSSNa polymer at various temperatures from 293 to 443 K. Insets show photographs from top to bottom taken after PSSNa polymer excited by 365 nm at 293, 348, 373, 418, and 443 K

To validate our hypothesis, we selected a poly(styrene sulfonic acid) sodium (PSSNa) (*M*_w_ = 80,000) as an ionic polymer model (Fig. 2a). In PSSNa polymer, aromatic phenyls acted as the chromophore, meanwhile sulfonate sodium substituents as locks were used to restrict the chromophore motions. Under the irradiation at a 365 nm UV lamp, PSSNa polymer showed sky-blue emission with a photoluminescence quantum yield (PLQY) of 5.3% in solid state at room temperature. At 77 K, it increased to 10.8% (Supplementary Table 1). After the removal of excitation source, yellow ultralong phosphorescence was observed by naked eyes, which lasted for several seconds under ambient conditions (Fig. 2a and Supplementary Movie 1). This phenomenon was distinctly different from the traditional polymer, polystyrene (PS), which revealed only fluorescence excited by 280 nm with a short lifetime of 4.82 ns (Supplementary Fig. 14 and Table 2), and no phosphorescence signal was detected under ambient conditions. These results suggested that the ionic cross-linking of sulfonate sodium substituents played a critical role in ultralong phosphorescence in the PSSNa polymer. Unexpectedly, with variation of excitation wavelength, the persistent luminescence showed an obvious redshift with main peaks changing from 540 to 560 nm (Supplementary Table 3), as shown in Fig. 2b and Supplementary Figure 16. To the best of knowledge, the excitation dependent persistent luminescence was rarely investigated in polymer system. Time-resolved emission spectra of dry PSSNa polymer excited at 325 nm further revealed stability of LRTP over time (Fig. 2c). With the delay of time, the profiles of phosphorescence spectra kept stable. As shown in Fig. 2d, it was found that the lifetimes of emission bands at 540 and 560 nm were up to 894 and 463 ms, indicating the LRTP nature. We speculated that the different lifetimes of phosphorescence emission may be ascribed to different excited states from different aggregations between chromophores. In a further set of experiments, we investigated the influence of temperature on long-lived phosphorescence. Remarkably, the range of color variation was extended from blue to orange at 77 K (Supplementary Fig. 16), corresponding phosphorescence peaks red-shifted from 441 to 568 nm (Fig. 2e). From Fig. 2f, it was found that the phosphorescence intensity gradually decreased with temperature increasing. Unexpectedly, phosphorescence signals could still be detected when PSSNa polymer was heated up to 443 K (Fig. 2f), owing to strong ionic bonding interactions. It was noted that no phosphorescence can remain at such a high temperature in the previously reported organic systems.

### Influence of different ions on LRTP

**Fig. 3:**
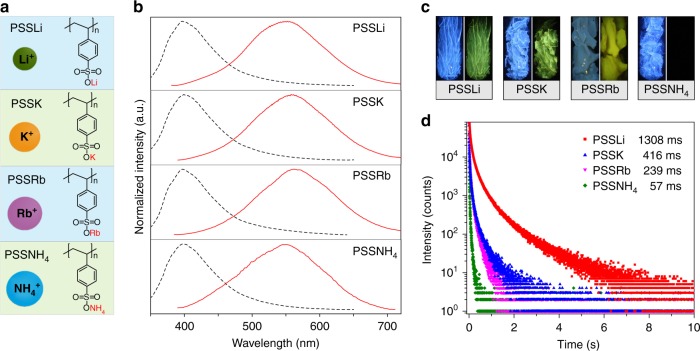
Photoluminescence investigation of polymers with different ion linkers in solid state under ambient conditions. **a** Chemical structures of PSSLi, PSSK, PSSRb, and PSSNH_4_ polymers. **b** Steady-state photoluminescence (dash lines) and phosphorescence (solid lines) spectra of PSSLi, PSSK, PSSRb, and PSSNH_4_ polymers, respectively. **c** Photographs of PSSLi, PSSK, PSSRb, and PSSNH_4_ polymers taken before (left) and after (right) turn-off of a 365 nm UV lamp. **d** Lifetime profiles of the phosphorescence emission bands at 550 nm for PSSLi, PSSK, PSSRb, and PSSNH_4_ polymers excited by 365 nm

In a further set of experiments, we investigated the influence of different ions on ultralong organic phosphorescence in polymers. After replaced with different cations (Li^+^, K^+^, Rb^+^, NH_4_^+^) (Fig. 3a), we found that the profiles of both steady-state PL and phosphorescence spectra of ionic polymers were similar with emission peaks at around 397 and 550 nm (Fig. 3b, Supplementary Fig. 18 and Tables 2, 4), respectively. Similar to PSSNa polymer in the solid state, blue fluorescence and yellow ultralong phosphorescence could be observed for these ion modified polymers by naked eyes under 365 nm lamp on and off, except for PSSNH_4_ (Fig. 3c and Supplementary Movie 2). As shown in Fig. 3d, with ionic radius increasing, the LRTP lifetimes gradually decreased from 1308 to 57 ms, possibly ascribing to quenching effect by heavy atoms or more intensive non-radiative transitions by weaker interactions between benzenesulfonates. Therefore, the LRTP was too weak to be observed for PSSNH_4_ polymer.

To explore the influence of ions with different charges on URTP, we synthesized a set of polymer phosphors with Mg^2+^, Ca^2+^, Al^3+^, and Gd^3+^ ions, namely, PSSMg, PSSCa, PSSAl, and PSSGd, respectively. Impressively, all polymer phosphors showed bright ultralong phosphorescence after the excitation source (a 365 nm UV lamp) was switched off (Supplementary Fig. 19 and 20). As shown in Supplementary Fig. 21–23, PSSMg, PSSCa, and PSSGd polymers showed yellow ultralong phosphorescence with lifetimes of 1152, 845, and 315 ms, respectively. For PSSAl polymer, it showed green ultralong phosphorescence with a lifetime of 765 ms (Supplementary Fig. 21 and 23). Compared with PSSNa phosphor, both PSSMg and PSSAl polymer phosphors showed longer phosphorescence lifetimes, which might be ascribed to stronger ionic bonding interactions for high charged ion. Impressively, the LRTP lifetimes decreased from PSSMg (1152 ms) to PSSCa (845 ms) and from PSSAl (765 ms) to PSSGd (315 ms), which stressed the fact that the LRTP lifetimes will decrease with ionic radius increasing. Taken together, we speculated that the large ionic radius is harmful to the prolonging of LRTP lifetime, whereas the high ion charge state is beneficial. Therefore, the LRTP lifetime can be managed by balancing the ionic radius and charge state.

### Proposed mechanism for long-lived phosphorescence

**Fig. 4:**
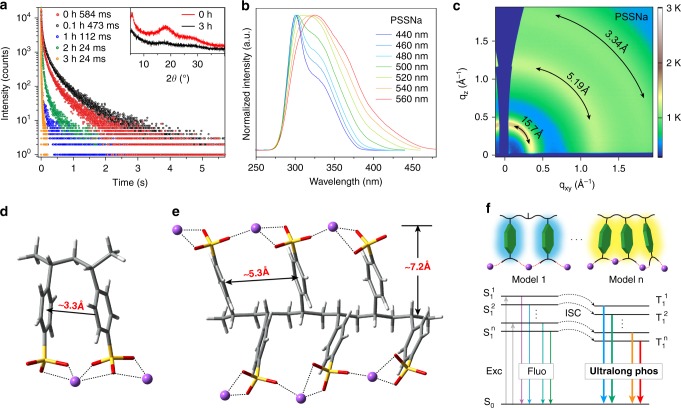
A plausible mechanism for ultralong phosphorescence in polymers. **a** The lifetime profiles of PSSNa in solid monitoring at 550 nm excited by 325 nm after exposed to air (humidity: 55%) for different periods of time. Inset shows the PXRD patterns of PSSNa in solid exposed for 0 and 3 h. **b** The excitation spectra of PSSNa polymer monitoring 440, 460, 480, 500, 520, 540, and 560 nm at 77 K. **c** 2D GI-WAXS pattern of dry PSSNa film. Optimized molecular structures of PSSNa models containing (**d**) two units and (**e**) six units by DFT. **f** Proposed mechanism for multicolor ultralong phosphorescence as the excitation wavelength changed. Noted that the schematic illustration of Model 1 and n represent the isolated and the aggregated chromophores in polymers, respectively

To gain deeper insight into the underlying mechanism for ultralong phosphorescence in ionic polymers, a set of control experiments were conducted. As shown in Fig. 4a, the lifetimes of ultralong phosphorescence gradually decreased when PSSNa film was exposed to wet air (humidity: 55%) from 0 to 3 h. It was found that the crystalline degree decreased after the PSSNa film was exposed in air for 3 h, demonstrating the moisture can partially destroy the ionic bonding to tune the LRTP lifetime^37^. Compared with the moisture, oxygen had slight effect on phosphorescence lifetimes owing to barrier of the dense aggregation and slow diffusion of oxygen in dense film, leading to a little decrease in the phosphorescence intensity and lifetime at *t* = 0 h (Supplementary Figs. 24–26). In a word, ionic bonding plays a significant role in generating ultralong phosphorescence. Moreover, from Supplementary Table 5, it easily found that the non-radiative decay rates were at least one order of magnitude higher than radiative decay rates, indicating the non-radiative decay rates played a dominant role in manipulating LRTP in ionic polymer phosphors. The suppression of non-radiative transitions for LRTP was also confirmed by the energy gap law with PSSNa phosphor as a model^38,39^ (Supplementary Fig. 25). Notably, compared with PSS polymer without ion substitution, the non-radiative decay rates for most ionic polymer phosphors were smaller, which further proved that the ions played a vital role in suppressing non-radiative transitions for LRTP.

To further probe the origin of excitation-dependent long-lived phosphorescence, we first investigated the excitation spectra of PSSNa. By monitoring different phosphorescence emission bands, the excitation spectra showed obvious redshift at both room temperature and 77 K (Fig. 4b and Supplementary Fig. 17), indicating there existed different aggregates in charge of colorful phosphorescent emission. The existence of different aggregates was further experimentally confirmed by concentration-dependent phosphorescence on a model monomer (4-vinylbenzenesulfonic acid sodium) at 77 K (Supplementary Fig. S30). With the monomer concentration increasing, phosphorescent emission bands were gradually redshifted from 450 to 555 nm owing to forming different ground state aggregates at different molecular concentration (Supplementary Fig. 30). From Grazing-incidence wide-angle X-ray scattering (GI-WAXS) patterns (Fig. 4c) and the corresponding scattering profiles in the in-plane and out-of-plane directions (Supplementary Fig. 32), we found that there existed three peaks, corresponding to three types of chromophore arrangements in dry PSSNa film. The first peak at low *q* (0.41 Å^−1^) was attributed to inter-polymer chain packing (15.7 Å), and the other two peaks at higher *q* = 1.22 and 1.87 Å^−1^ corresponded to *d*-spacing of 5.19 and 3.34 Å, which were attributed to sodium benzenesulfonate triad spacing along one polymer chain^40^. These results were consistent with optimized stacking of the chromophores in polymer chains calculated by density functional theory (DFT) (Fig. 4d, e). Taken together, we speculated that the long-lived phosphorescence in polymers was stemmed from the stabilization of triplet excitons by the aggregation of adjacent benzene units with short distances at around 3.34 Å at room temperature. The variation of long-lived phosphorescence might be ascribed to the different aggregates of adjacent benzene units, like the Model n in Fig. 4f. Owing to the restriction of molecular motions at 77 K, the multiple channels of phosphorescence emission as shown by the Model 1 to Model n were unlocked (Supplementary Fig. 33), thus leading to the excitation dependent colorful ultralong phosphorescence.

To clarify the formation of triplet states for long-lived emission, we studied the dynamics of triplet generation by the transient absorption (TA) experiments on the PSSNa polymer film (Supplementary Fig. 34). As shown in the nanosecond (ns)-resolved TA data, excited-state absorption (ESA) features are observed ranged from 360 to 700 nm (Supplementary Fig. 34a). A spectral transfer to a long persistent ESA component centered at 440 nm is observed with a characteristic lifetime of ~ 6 ns. The new generated excited state can be safely assigned to the triplet excited state considering its long lifetime of over 100 μs. Moreover, we studied the excitation-density-dependence of triplet generation and found that the kinetics was nearly insensitive to the pump power (Supplementary Fig. 34b, c), which suggests that the triplet states are formed from an ISC process rather than the bimolecular recombination following exciton dissociation. We further confirm the assignment by the global fitting algorithm to analyze the broadband TA signal recorded at different pump fluences, and find out that the lifetimes of spectral transfer are independent of pump fluence (Supplementary Fig. 34d–34f). The existence of triplet states in PSSNa phosphors was experimentally confirmed by photodegradation of anthracene-9,10-diyl-bis-methylmalonate (ADMA), a chemical tracker of singlet oxygen (Supplementary Fig. 35).

### Universality confirmation of the ionic interlocking for LRTP

**Fig. 5:**
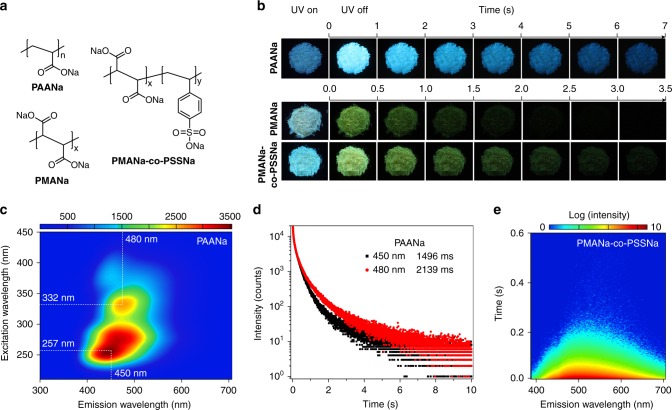
Photophysical properties of PAANa, PMANa, and PMANa-co-PSSNa polymers in solid state under ambient conditions. **a** Molecular structures of PAANa, PMANa, and PMANa-co-PSSNa. **b** Photographs of PAANa, PMANa, and PMANa-co-PSSNa polymers taken before and after the irradiation of a 310 nm UV lamp. **c** Excitation-phosphorescence emission mapping of the PAANa polymer. **d** The lifetime profiles of the PAANa polymer monitoring at 450 and 480 nm. **e** Time-resolved emission spectra of the PMANa-co-PSSNa copolymer excited by 375 nm

To test the universality of our approach, we introduced the ionic bonding cross-linking into nonaromatic polymers, then designed a set of the ionic polymers, namely PAANa, PMANa, and PSSNa-co-PMANa (Fig. 5a). With chromophore variations, the LRTP emission colors were successfully tuned from yellow to blue (Fig. 5b). Like PSSNa polymer, these ionic polymers also showed excitation-dependent ultralong phosphorescence under ambient conditions (Fig. 5c and Supplementary Figs. 36–40). The blue LRTP band showed a record lifetime of 1496 ms monitoring the emission band at 450 nm excited by 254 nm. Unexpectedly, the lifetime of LRTP emission band at 480 nm was up to 2139 ms (Fig. 5d). To the best of our knowledge, it is the longest lifetime of organic phosphorescence in polymer luminogens (Supplementary Fig. 41). Carboxylate (-COO-) acted as the chromophore was restricted by ionic bonding, thus contributing to ultralong phosphorescence of PAANa polymer. PMANa polymer containing carboxylate (-COO-) and sodium ion also demonstrated ultralong phosphorescence excited by 310 nm with lifetime of 378 ms (Fig. 5b). Besides, ionic copolymer PMANa-co-PSSNa polymer in the solid state can emit yellow-green ultralong phosphorescence, which can be observed by naked eye for several seconds (Fig. 5b), with the lifetime of 385 ms monitoring at the peak at 500 nm. Time-resolved emission spectra of PMANa-co-PSSNa films exhibited that the peaks of phosphorescence spectra shifted from 500 to 530 nm (Fig. 5e) with the time delayed. Therefore, ionic bonding cross-linking can be also suitable to ionic copolymers with LRTP nature.

## Discussion

In summary, we have developed a facile and concise chemical strategy to achieve long-lived organic phosphorescence among traditional amorphous polymers. With an intense intermolecular subunit interlocking by cations in polymers, a record LRTP lifetime of up to 2.1 s was obtained under ambient conditions. Besides, the long-lived phosphorescent emission can be controllably tuned by manipulation of excitation wavelength. More importantly, the universality of our approach was also proved in non-aromatic polymers. Taken experimental and theoretical studies together, we proposed that the cross-linking between chromophores with ionic bonding played a critical role in suppressing non-radiative transitions for LRTP enhancement. This finding not only expands the scope of metal-free organics with LRTP nature, but also paves a way to study ultralong phosphorescent materials for potential application in polymer-based flexible electronics.

## Methods

### Reagents and materials

PSSNa ((poly(sodium 4-styrenesulfonate)) (average *M*_w_ = 80,000) solution was purchased from Shanghai macklin Biochemical Co., Ltd. PMANa-co-PSSNa powder (Poly(maleic acid-co-4-styrenesulfonic acid) sodium salt)) (the mole ratio of PSSNa and PMANa is 1:1, average *M*_w_ = 20,000) was purchased from Sigma-Aldrich Co. Ltd. They purified by a dialysis bag (mwco 3500). PAA (polyacrylic acid) (*M*_w_ = 40,000 ~ 60,000) powder and PMA (polymaleic acid) 50% in water were purchased from Shanghai macklin Biochemical Co., Ltd. Lithium hydroxide, sodium hydroxide, potassium hydroxide, rubidium hydroxide, ammonium hydroxide, magnesium hydroxide, aluminum hydroxide, gadolinium oxide, calcium oxide, and PSS (polystyrene sulfonic acid) (*M*_w_ = 75,000) solution used in the experiments were purchased from commercial sources without further purification.

### Measurements

Nuclear magnetic resonance (^1^H NMR) spectra were obtained on a Bruker Ultra Shield Plus 400 MHz spectrometer. Chemical shift was relative to tetramethylsilane (TMS) as the internal standard. UV-visible absorption spectra were obtained using Shimadzu UV-1750. Steady-state fluorescence/phosphorescence spectra and excitation spectra were measured using Hitachi F-4600. The lifetime and time-resolved emission spectra were obtained on Edinburgh FLSP920 fluorescence spectrophotometer equipped with a xenon arc lamp (Xe900), a nanosecond hydrogen flash-lamp (nF920), a microsecond flash-lamp (μF900), respectively. The luminescent photos and videos were taken by a Cannon EOS 700D camera at room temperature. Scanning electron microscope (SEM) images and energy-dispersive X-ray spectroscopy (EDS) mapping were collected by scanning electron microscope (JSM-7800F). GIWAXS measurements were conducted using the SAXS/WAXS beamline of the Australian Synchotron. The polymer films were spin-coated using the same preparation method as for the device active layer on the substrates of cleaned silicon wafers. Samples were analyzed using an X-ray energy of 11 keV and incident angles ranging from *Ω* = 0.02 to 0.35 in 0.005 increments, which allowed signal optimization near the critical angle of the polymer film, but below the critical angle of the substrate. Data from GIWAXS experiments were analyzed using a customized version of NIKA 2D based in IgorPro software. FI-IR spectra were collected by NICOLET iS50 FT-IR. Absorption spectra of ADMA were obtained by PE Lambda 950. X-ray crystallography was achieved using a Bruker SMART APEX-II CCD diffractometer with graphite monochromated Mo-Kα radiation.

### Transient absorption spectroscopy

For ns-resolved TA measurement, we employ a pump laser of a frequency-tripled sub-nanosecond laser (Picolo AOT MOPA, InnoLas) at 355 nm (pulse duration ~ 0.8 ns). The probe beam is a broadband supercontinuum light source generated by focusing a small portion of the femtosecond Ti:Sapphire laser beam (Libra, Coherent Inc.) onto a 5-mm-thick CaF_2_ plate. The lasers are synchronized to the probe pulse with a desired delay by an electronic delay generator (SRS DG645, Stanford Research System). The TA signal is then analyzed by a silicon CCD (S11071, Hamamatsu) mounted on a monochromator (Acton 2358, Princeton Instrument) at 1 kHz enabled by a custom-built control board from Entwicklungsbuero Stresing.

### Computational details

Several test segments from PSSNa polymer, including monomer, dimer, trimer, and hexamer, were constructed to probe the possible polymer configurations. The M062X functional has been proposed to rationally describe the weak intermolecular interactions (van der Waals and π–π coupling)^41^, and then was employed to optimize the ground-state structures of these chosen segments together with 6-31G(d) basis set, except for hexamer with 3-21G basis set. The excitation energies and natural transition orbitals for the singlet and triplet states of monomer were then evaluated by TD-DFT method. All the calculations were performed using Gaussian 09 program^42^. At the same level, the spin–orbit couplings between singlet ant triplet states were performed using PySOC code^43^.

## Supplementary information

Supplementary Information

Description of Additional Supplementary Files

Supplementary Movie 1

Supplementary Movie 2

## Data Availability

The data that support the findings of this study are available from the corresponding authors upon reasonable request.
