# Supplementary material for: Enabling long-lived organic room temperature phosphorescence in polymers by subunit interlocking
Source: Nat Commun. 2019 Sep 18;10:4247. doi: 10.1038/s41467-019-11749-x (PMC6751207; doi:10.1038/s41467-019-11749-x)
Supplement: Supplementary file 1 — Supplementary Information [file 41467_2019_11749_MOESM1_ESM.pdf]

# Supplementary Information

## Enabling long-lived organic room temperature phosphorescence in polymers by subunit interlocking

Suzhi Cai[1†], Huili Ma[1†], Huifang Shi[1†], He Wang[1], Xuan Wang[1], Leixin Xiao[2], Wenpeng Ye[1], Kaiwei Huang[1], Xudong Cao[1], Nan Gan[1], Chaoqun Ma[1], Mingxing Gu[1], Lulu Song[1], Hai Xu[1], Youtian Tao[1], Chunfeng Zhang[2], Wei Yao[1], Zhongfu An[1*], Wei Huang[1,3,4*]

*[1]Key Laboratory of Flexible Electronics & Institute of Advanced Materials, Nanjing Tech University, 30 South Puzhu Road, Nanjing 211816, China.*

*[2]National Laboratory of Solid State Microstructures, School of Physics, Collaborative Innovation Center for Advanced Microstructures, Nanjing University, Nanjing 210093, China.*

*[3]Institute of Flexible Electronics (IFE), Northwestern Polytechnical University (NPU), 127 West Youyi Road, Xi'an 710072, China.*

*[4]Key Laboratory for Organic Electronics and Information Displays & Institute of Advanced Materials, Jiangsu National Synergistic Innovation Center for Advanced Materials, Nanjing University of Posts and Telecommunications, Nanjing 210023, China.*

*[†]These authors contributed equally to this work.*

**Correspondence to: iamzfan@njtech.edu.cn; iamwhuang@nwpu.edu.cn*

## Supplementary Discussion

### Synthesis of PSSLi, PSSNa, PSSK, PSSRb and $PSSNH_4$ polymers

PSS solution (5 mL) was added to a 50 mL flask at room temperature. LiOH, NaOH, KOH, RbOH powder were dissolved in deionized water to form alkaline solution (2 mol/L), respectively. The alkaline solution or ammonia solution was dropped into PSS solution, regulating pH to slightly alkaline. The mixture was stirred for 4 h at room temperature, then poured into a dialysis bag (mwco 3500). The polymer was purified by dialysis against water for 3 days with a membrane (molecular weight cutoff, mwco 3500). PSSLi, $^1H$ NMR ($D_2O$): 7.41 (s, 2H), 6.51 (s, 2H), 1.34 (s, 3H); PSSNa, $^1H$ NMR ($D_2O$): 7.44 (s, 2H), 6.54 (s, 2H), 1.35 (s, 3H); PSSK, $^1H$ NMR ($D_2O$): 7.41 (s, 2H), 6.49 (s, 2H), 1.33 (s, 3H); PSSRb, $^1H$ NMR ($D_2O$): 7.48 (s, 2H), 6.59 (s, 2H), 1.39 (s, 3H); $PSSNH_4$, $^1H$ NMR ($D_2O$): 7.42 (s, 2H), 6.54 (s, 2H), 1.3 (s, 3H) (Supplementary Fig. 1).

### Synthesis of PSSMg, PSSAl, PSSGd, and PSSCa polymers

PSS solution (5 mL) and 25 mL ionized water was added to a 100 mL flask at room temperature. $Mg(OH)_2$ (1.5 g), Al $(OH)_3$ (1.5 g), $Gd_2O_3$ (1.5 g), CaO (1.5 g) powders were added into PSS solution respectively. The mixture was stirred until the pH value became neutral or alkaline. Then redundant powders were removed by centrifugation, and the solution was poured into a dialysis bag (mwco 3500). The polymer was purified by dialysis against water for 3 days with a membrane (molecular weight cutoff, mwco 3500). The structures of PSSMg, PSSAl, PSSGd and PSSCa were confirmed by energy-dispersive spectroscopy (EDS) spectra.

### Synthesis of PAANa and PMANa polymers

PAA powder (0.5 g) or PMA solution (5 mL) was dissolved in deionized water (50 mL) at a 100 mL flask at room temperature. NaOH solution (2 mol/L) was dropped into the solution, regulating pH to slightly alkaline. The mixture was stirred overnight at room temperature, then poured into a dialysis bag (mwco 3500). The polymer was purified by dialysis against water for 3 days with a membrane (molecular weight cutoff, mwco 3500).

Elementary mapping images of PSSNa polymer reveals the distribution of C, H, S, and Na elements (Supplementary Fig. 2). In addition, $Na^+$ cations uniformly distributed throughout the PSSNa polymer. Energy-dispersive spectroscopy (EDS) spectrum analyses on the mole ratio of S and Na ($\approx 1$) suggested that PSS had become PSSNa completely. Similarly, PSSK, PSSRb, $PSSNH_4$, PSSMg, PSSAl, PSSGd and PSSCa polymers were the products of PSS polymer ionized (Supplementary Figs. 3-5). Elementary mapping images of PAANa and PMANa polymers also demonstrated that $Na^+$ cations had been well distributed in polymers (Supplementary Figs. 6 and 7). Energy-dispersive spectroscopy (EDS)

spectrum analyses on the mole ratio of S and Na in PMANa-co-PSSNa polymer are about 1:5, suggesting 100% ionization (Supplementary Fig. 8). The PXRD of ionic polymer mentioned in this work exhibited the amorphous aggregation state (Supplementary Fig. 9).

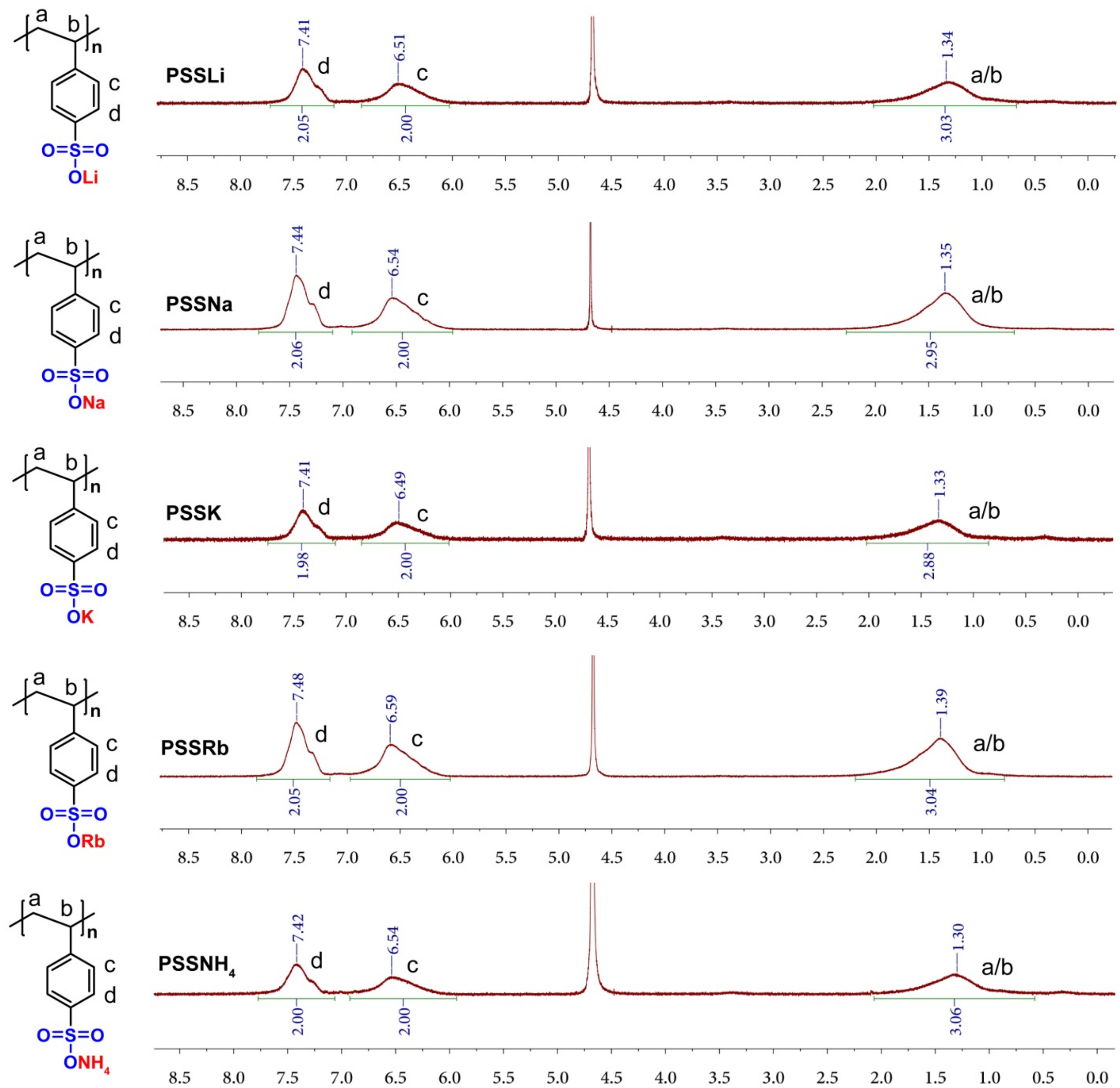


**Supplementary Figure 1. $^1$H NMR spectra of PSSLi, PSSNa, PSSK, PSSRb and $PSSNH_4$ in $D_2O$.**

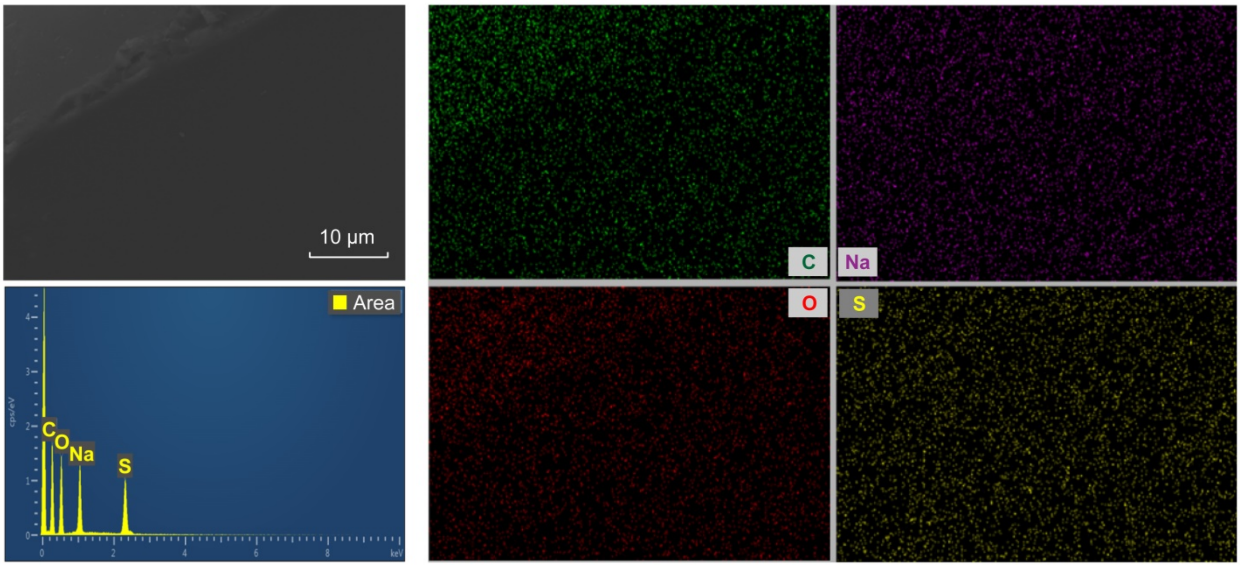


**Supplementary Figure 2. Energy-dispersive spectroscopy spectrum and elementary mapping images of PSSNa polymer.**

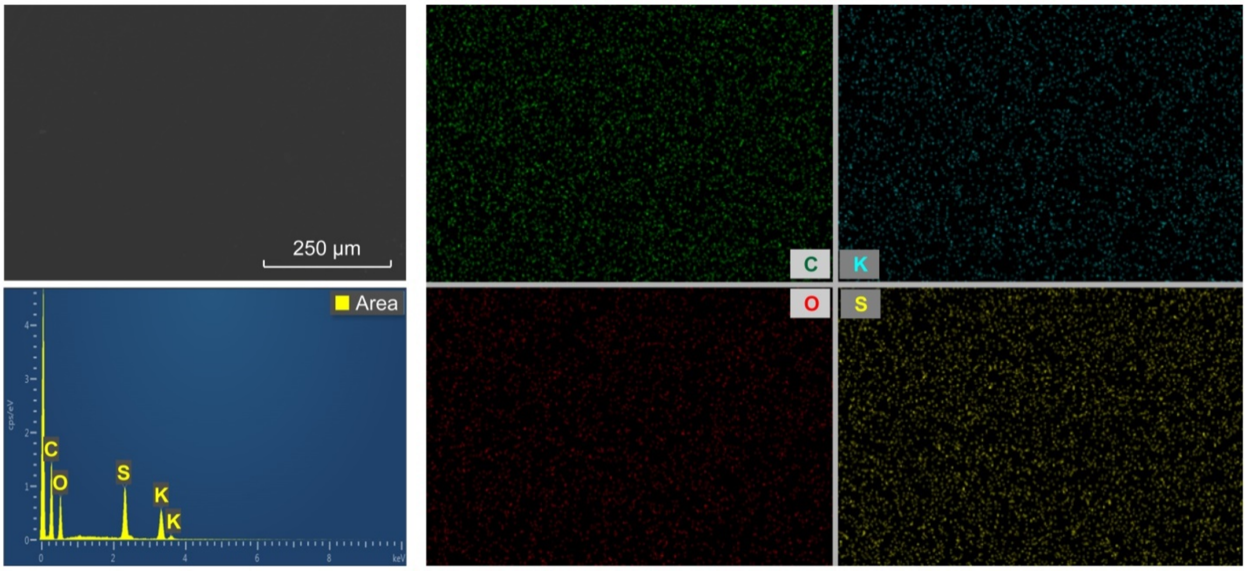


**Supplementary Figure 3. Energy-dispersive spectroscopy spectrum and elementary mapping images of PSSK polymer.**

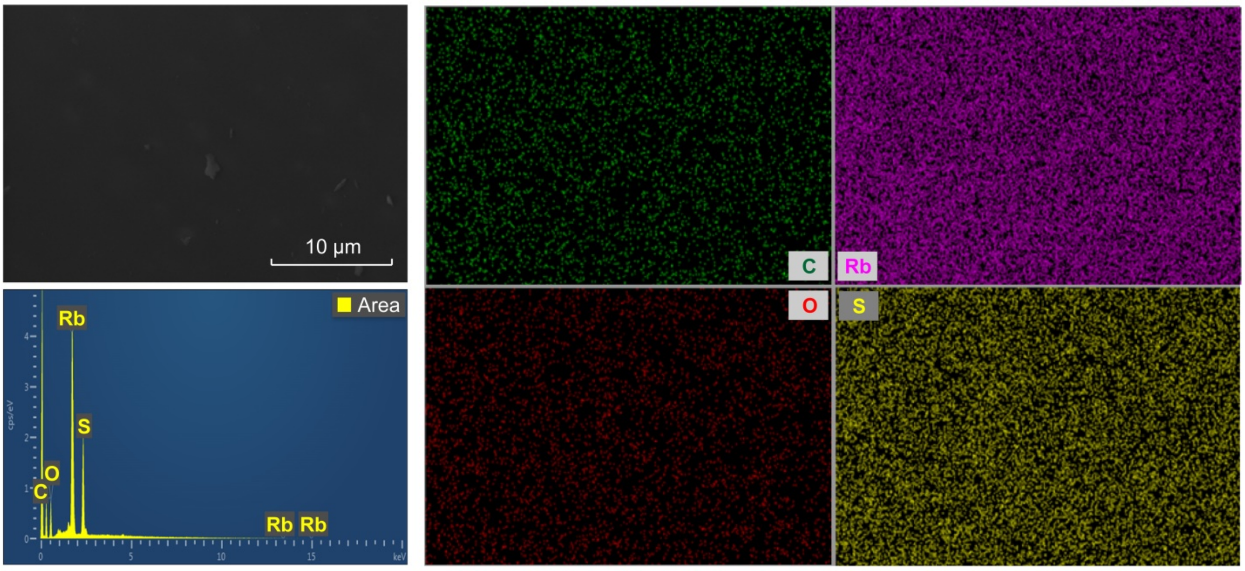


**Supplementary Figure 4. Energy-dispersive spectroscopy spectrum and elementary mapping images of PSSRb polymer.**

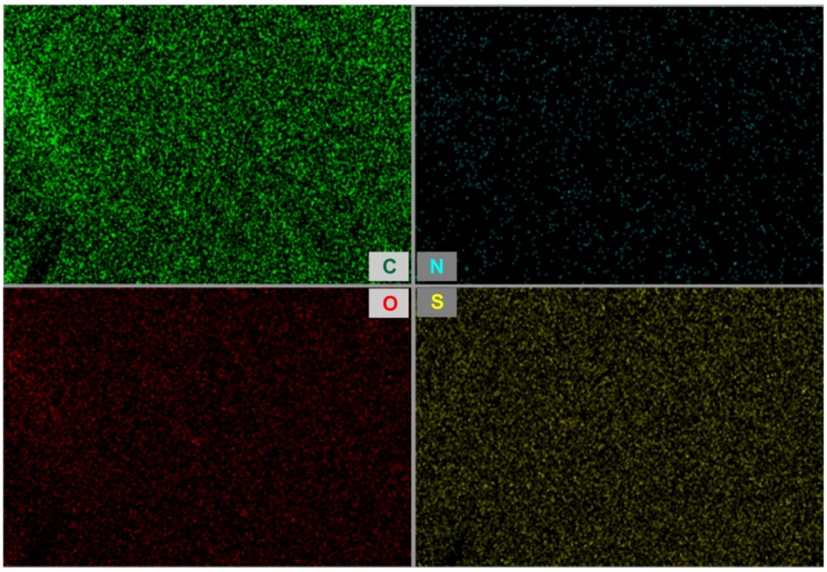


**Supplementary Figure 5. Energy-dispersive spectroscopy spectrum and elementary mapping images of $PSSNH_4$ polymer.**

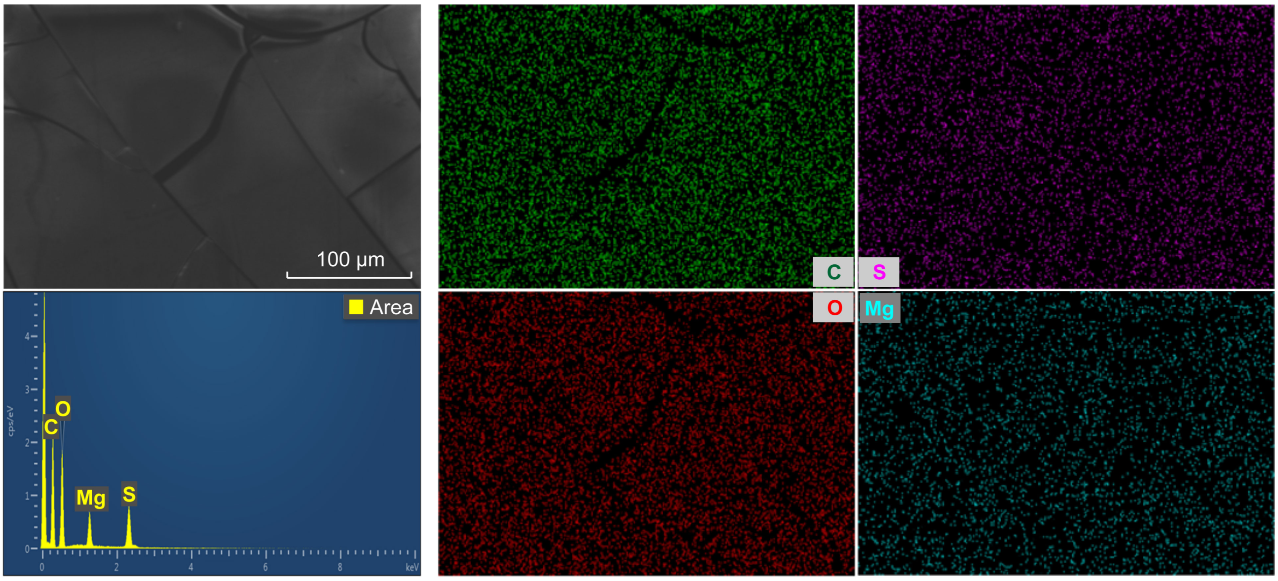


**Supplementary Figure 6. Energy-dispersive spectroscopy spectrum and elementary mapping images of PSSMg polymer.**

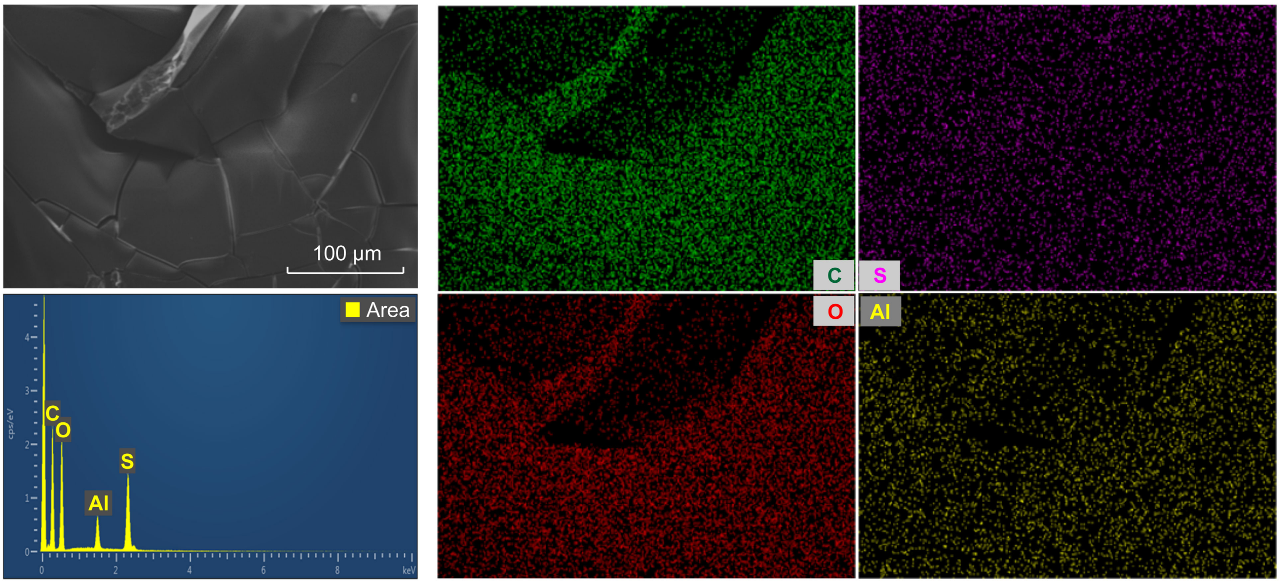


**Supplementary Figure 7. Energy-dispersive spectroscopy spectrum and elementary mapping images of PSSAl polymer.**

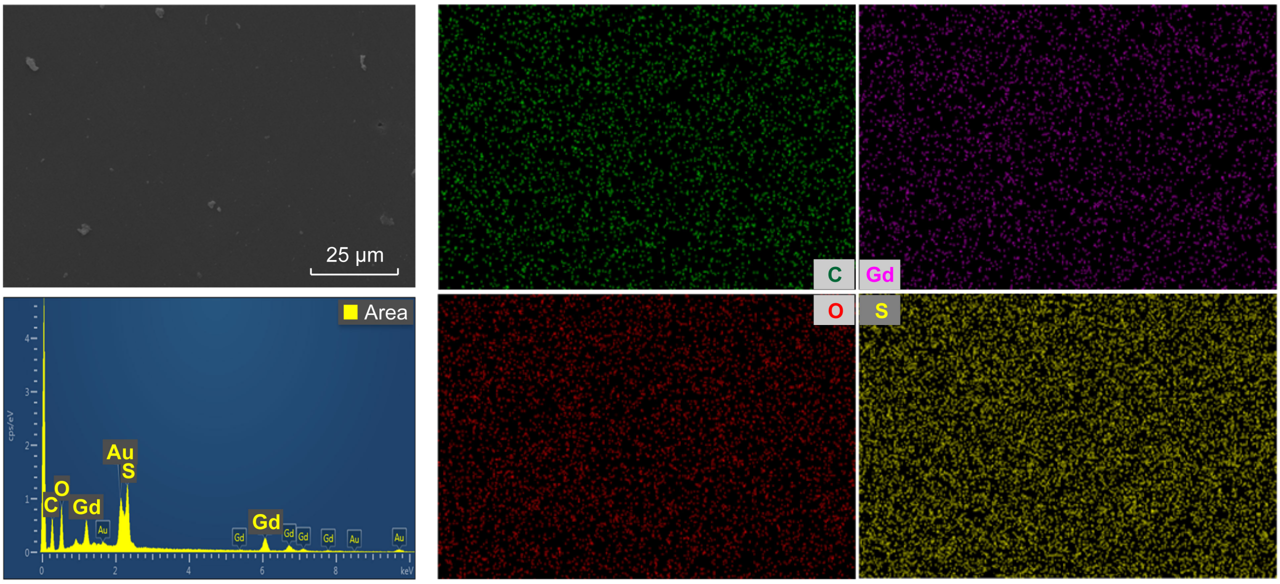


**Supplementary Figure 8. Energy-dispersive spectroscopy spectrum and elementary mapping images of PSSGd polymer.**

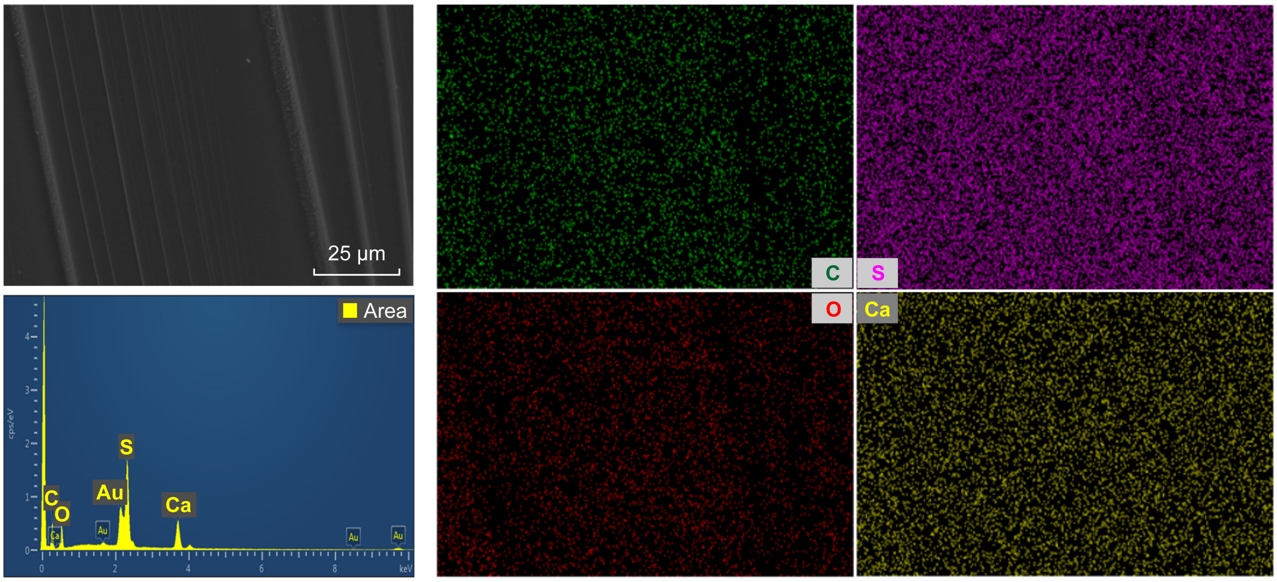


**Supplementary Figure 9. Energy-dispersive spectroscopy spectrum and elementary mapping images of PSSCa polymer.**

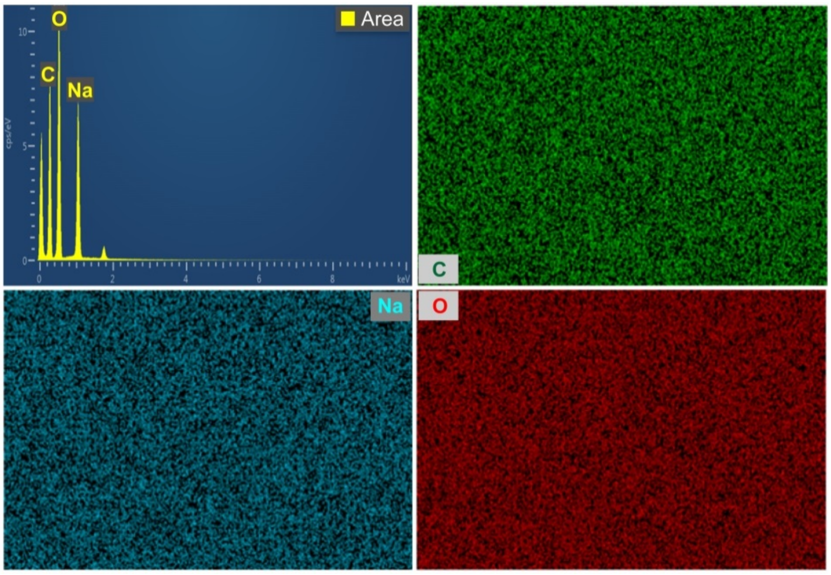


**Supplementary Figure 10. Energy-dispersive spectroscopy spectrum and elementary mapping images of PAANa polymer.**

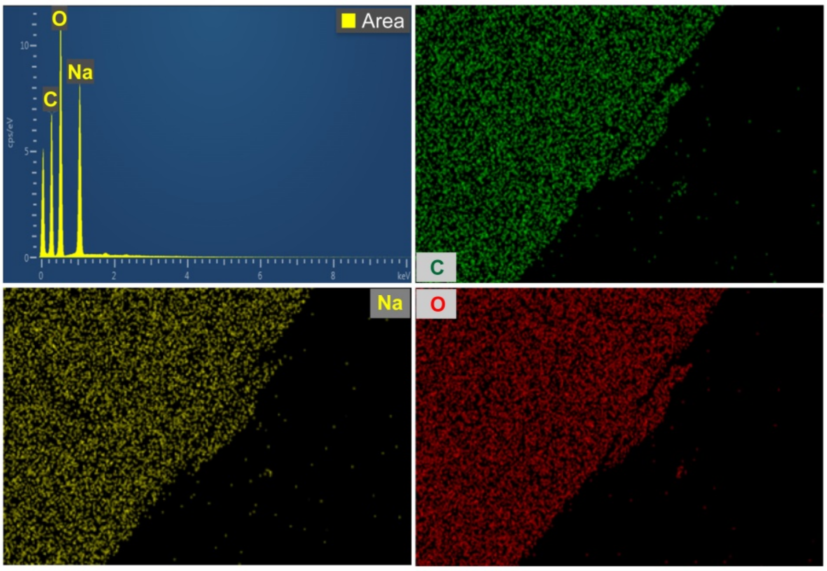


**Supplementary Figure 11. Energy-dispersive spectroscopy spectrum and elementary mapping images of PMANa polymer.**

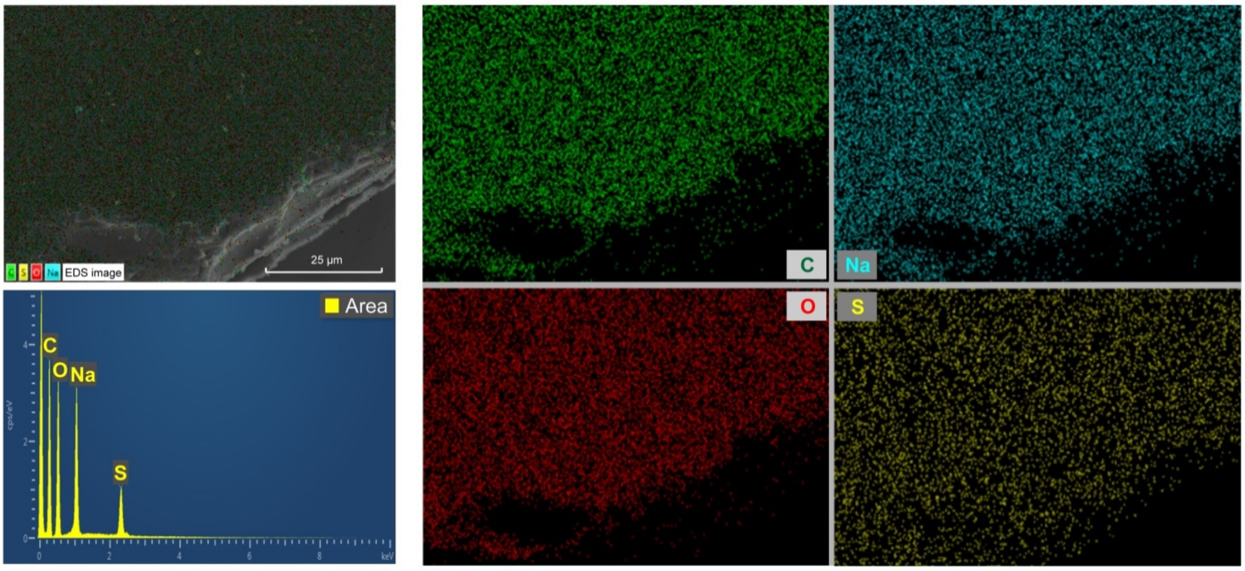


**Supplementary Figure 12. Energy-dispersive spectroscopy spectrum and elementary mapping images of PMANa-co-PSSNa polymer.**

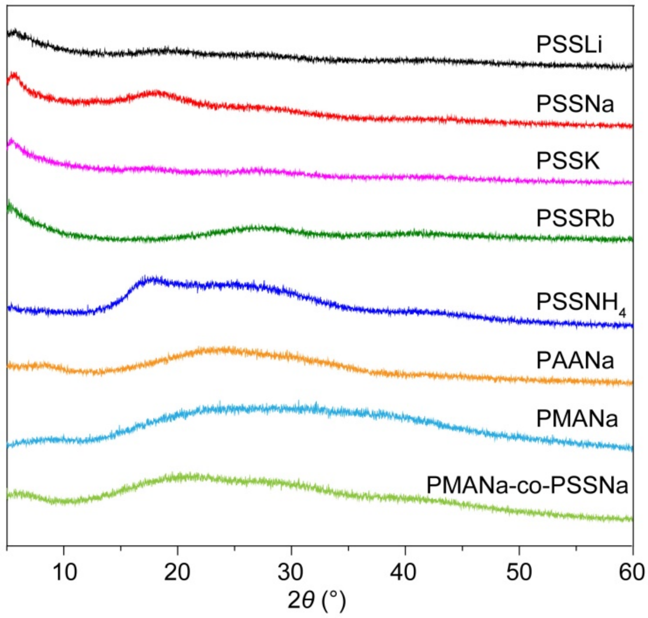


**Supplementary Figure 13. PXRD patterns of ionic polymers mentioned in this work.**

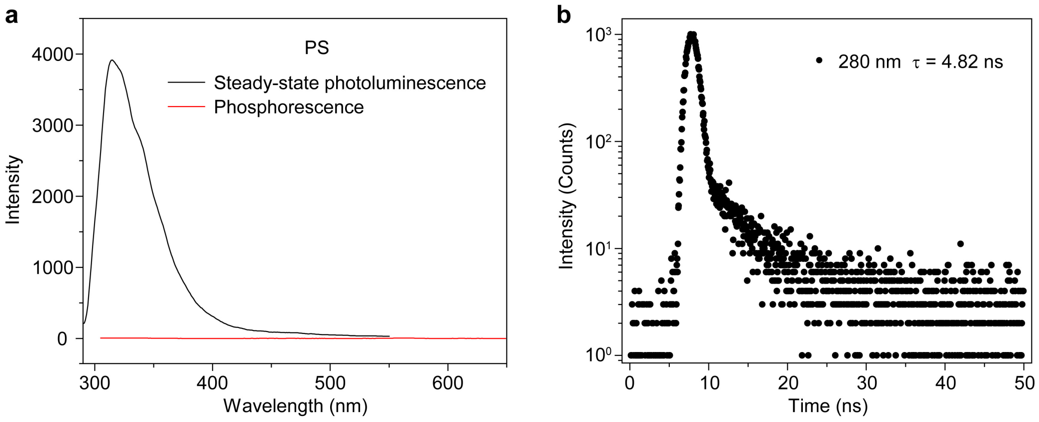


**Supplementary Figure 14. The photophysical properties of PS polymer in the solid state under ambient conditions. a,** Steady-state photoluminescence (black line) and phosphorescence (red line) spectra of PS in solid excited by 280 nm. **b,** Lifetime decay profile of PS polymer monitoring 280 nm excited at 280 nm.

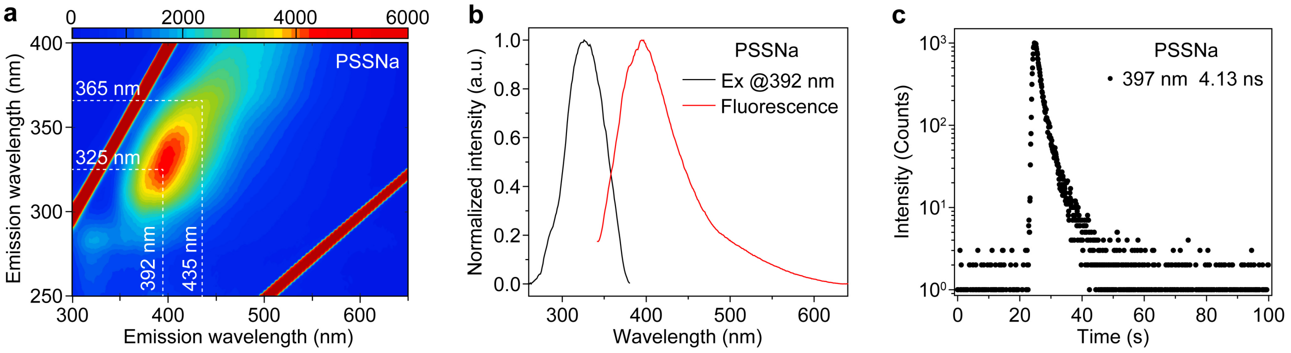


**Supplementary Figure 15. The photophysical properties of dry PSSNa polymer in the solid state under ambient conditions. a**, Excitation-photoluminescence mission mapping of PSSNa polymer. **b**, Steady-state photoluminescence spectrum (red line) of PSSNa polymer excited by 325 nm and the excitation spectrum (black line) of PSSNa polymer monitoring 392 nm. **c**, Lifetime decay profile of PSSNa polymer monitoring 397 nm excited at 325 nm.

The photoluminescence of PSSNa polymer showed the linely red-shifted with increasing excitation, which may be ascribed to different aggregates. The fluorescence emission peak of PSSNa polymer was at 392 nm (4.23 ns) excited by 325 nm (Supplementary Fig. 15).

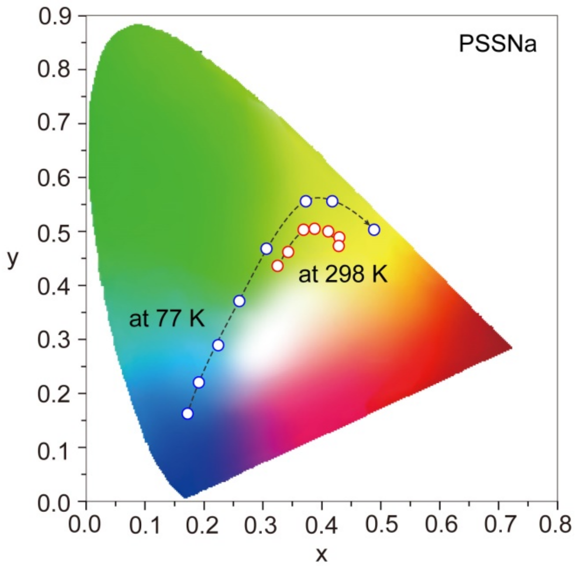


**Supplementary Figure 16. CIE chromaticity coordinate variation of phosphorescence emission color of PSSNa along with excitation wavelength.** Note that red and blue dots represent the CIE coordination of UOP for PSSNa polymer at 298 K and 77 K, respectively.

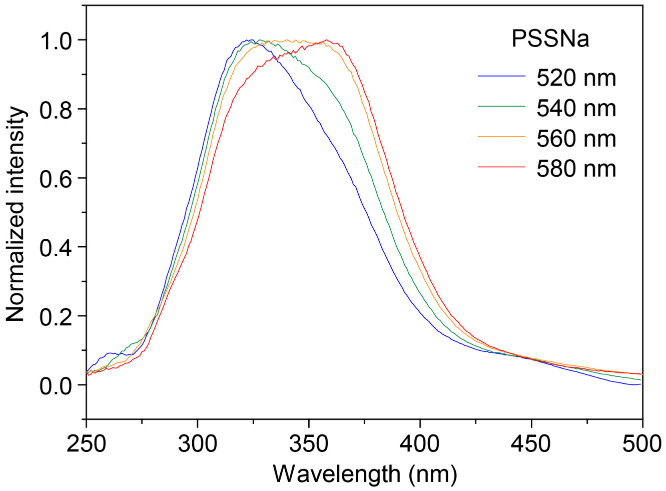


**Supplementary Figure 17. Excitation spectra of PSSNa polymer in the solid-state monitoring 520, 540, 560 and 580 nm at 298 K.**

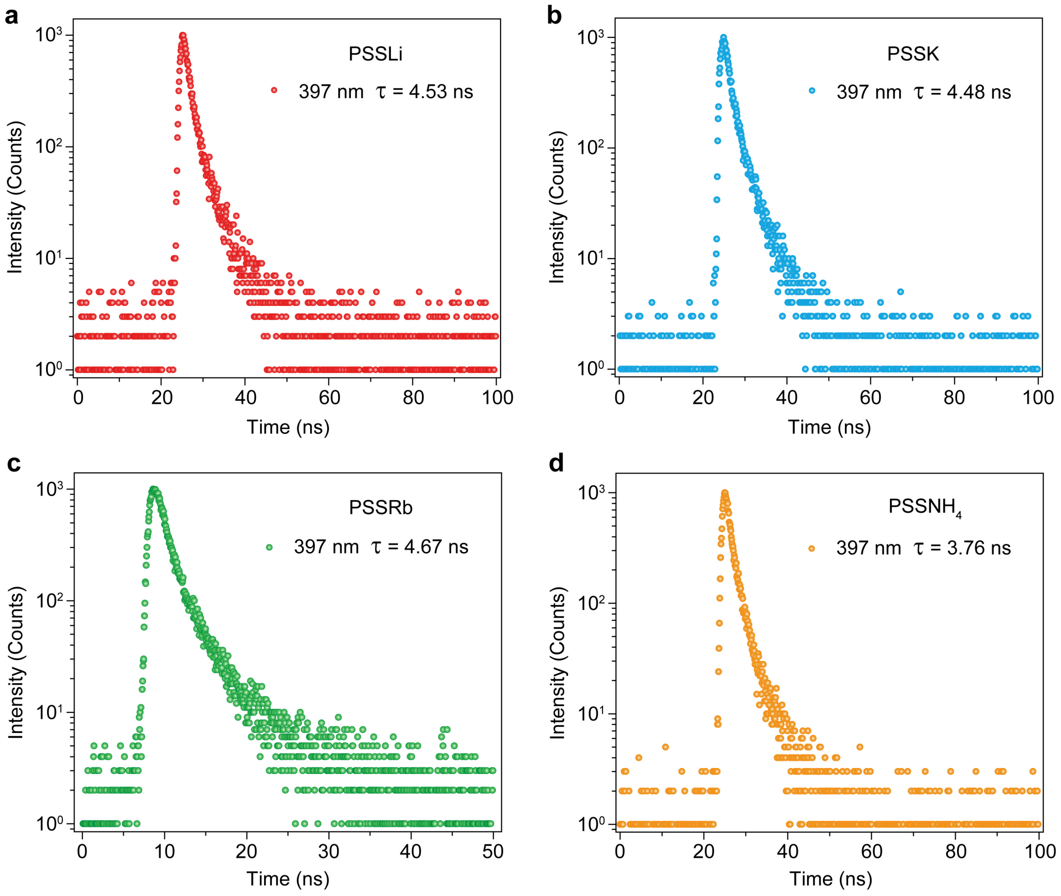


**Supplementary Figure 18. Lifetime decay profiles of the fluorescence emission bands of (a) PSSLi, (b) PSSK, (c) PSSRb and (d) $PSSNH_4$ polymers under ambient conditions.**

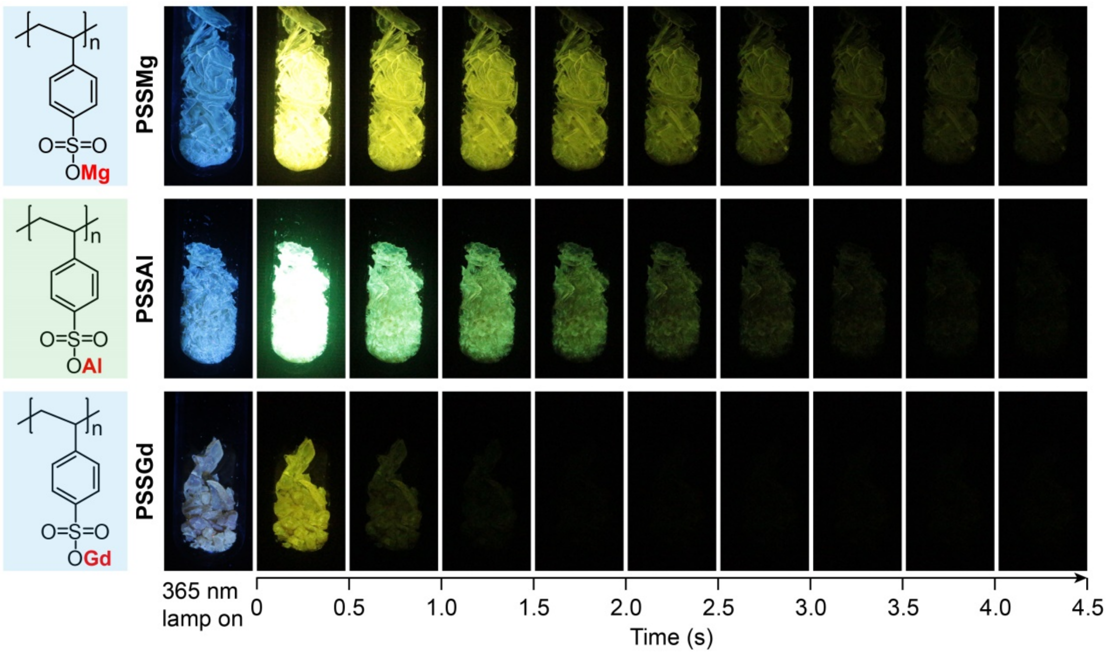


**Supplementary Figure 19. Chemical structures of PSSMg, PSSAl and PSSGd polymers, and photographs of PSSMg, PSSAl and PSSGd polymers taken before and after the irradiation of a 365 nm UV lamp under ambient conditions.**

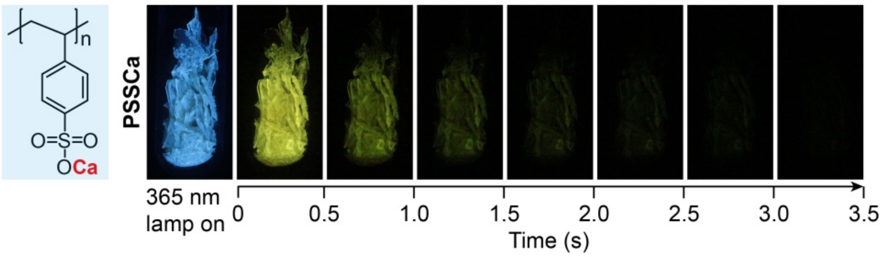


**Supplementary Figure 20. Chemical structure of PSSCa polymer and photographs of PSSCa polymer taken before and after the irradiation of a 365 nm UV lamp.**

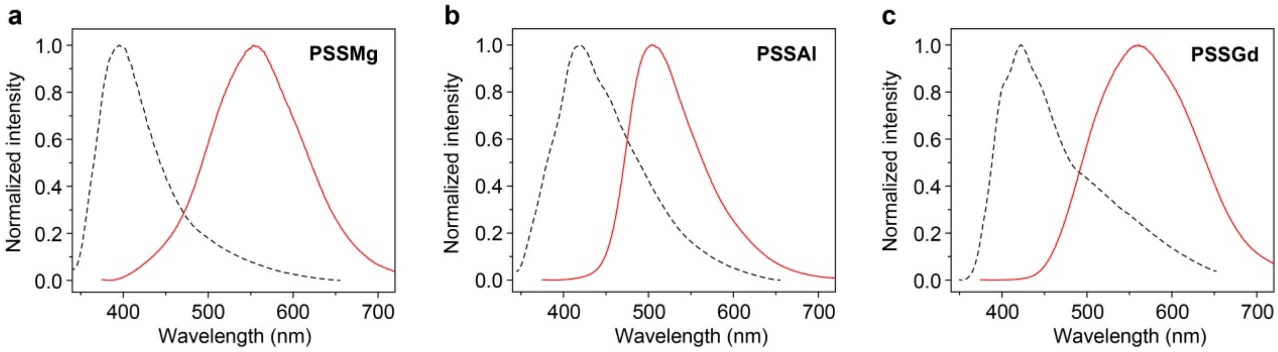


**Supplementary Figure 21. Steady-state photoluminescence (dash line) and phosphorescence (solid line) spectra of PSSMg (a), PSSAl (b) and PSSGd (c) polymers under ambient conditions.**

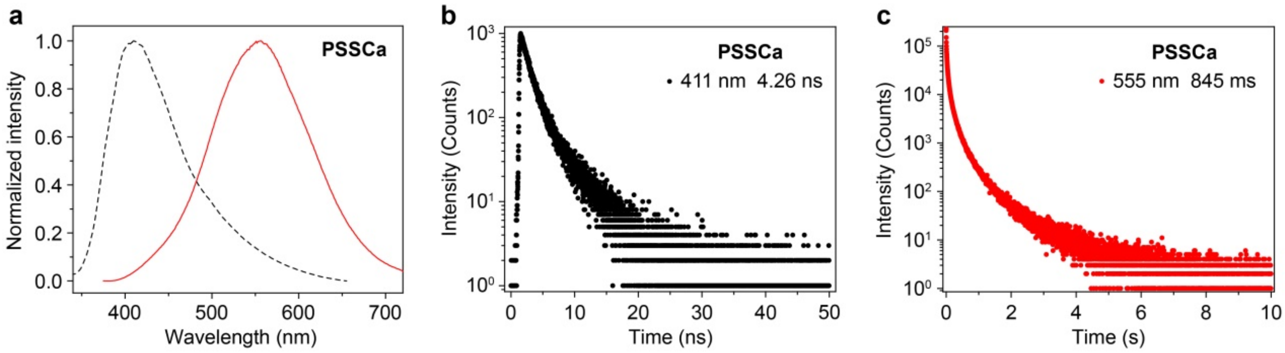


**Supplementary Figure 22. The photophysical properties of dry PSSCa polymer in the solid state under ambient conditions.** **a**, Steady-state photoluminescence (dash line) and phosphorescence (solid line) spectra of PSSCa polymer under ambient conditions. **b**, Lifetime decay profile of PSSCa polymer monitoring at 411 nm excited by 330 nm. Photographs of PSSCa polymer taken before and after the irradiation of a 365 nm UV lamp. **c**, Lifetime profiles of the phosphorescence emission bands at 555 nm for PSSCa polymer excited by 365 nm.

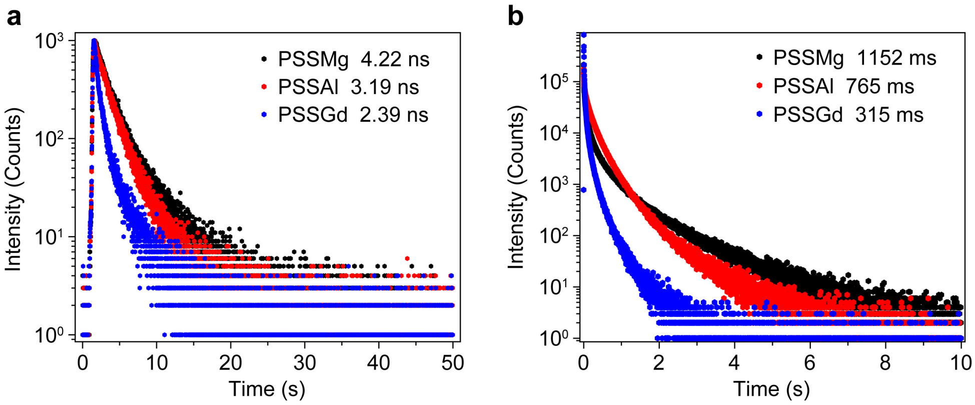


**Supplementary Figure 23. a,** Lifetime decay profiles of PSSMg, PSSAl and PSSGd polymers excited at 330 nm under ambient conditions. **b**, Lifetime decay profiles of PSSMg, PSSAl and PSSGd polymers excited at 365 nm under ambient conditions.

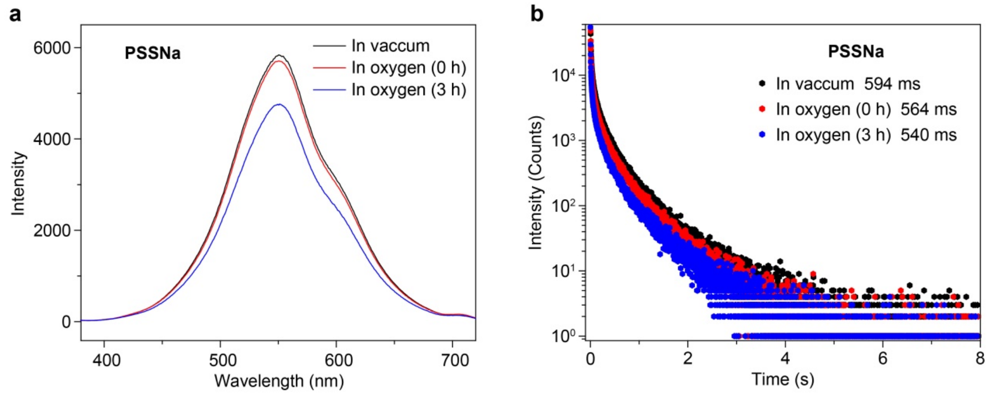


**Supplementary Figure 24. The influence of oxygen on phosphorescence emission of PSSNa polymer at room temperature. a,** Phosphorescence spectra of the PSSNa polymer in vacuum and oxygen for 0 and 3 h. **b,** Lifetime profiles of PSSNa polymer under the vacuum and after exposure to oxygen for 0 and 3 h.

When PSSNa films was exposed to oxygen, the emitters on the surface were promptly quenched by oxygen. Then oxygen can gradually diffuse into dense film. More and more emitters were gradually quenched, thus leading to time dependent influence of oxygen on phosphorescent performance for PSSNa film. This is an effect on the photophysics. To exclude the photodegradation during oxygen exposure, a set of control experiments including fourier transform infrared (FTIR) spectroscopy and $^1$H NMR was conducted. PSSNa polymer was treated in three ways: placed in vacuum and exposed to oxygen for 3 hour as well as irradiated by a 365 nm lamp for 5 min in oxygen atmosphere. From Supplementary Figure 25 and 26, it was found that the FTIR and NMR spectra were identical, indicating there was no photodegradation for PSSNa polymer during exposure to oxygen. Taken these results together, we concluded that this is an effect on the photophysics rather than photodegradation.

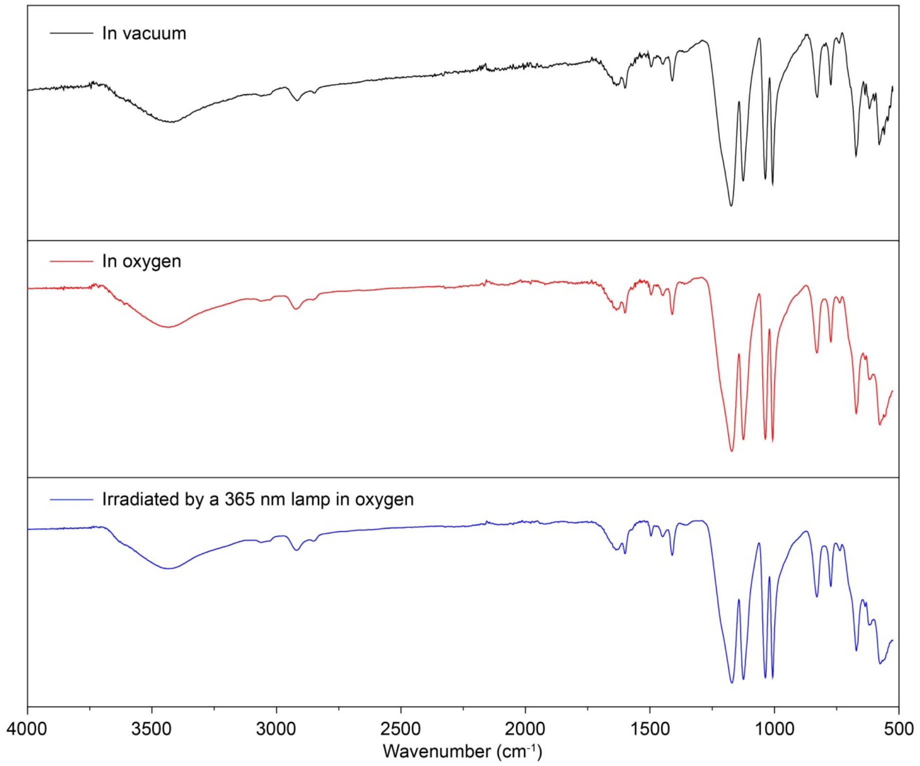


**Supplementary Figure 25.** Fourier transform infrared (FTIR) spectroscopy of PSSNa polymer in vacuum, in oxygen for 3 hours or irradiated by a 365 nm lamp for 5 min in oxygen, respectively.

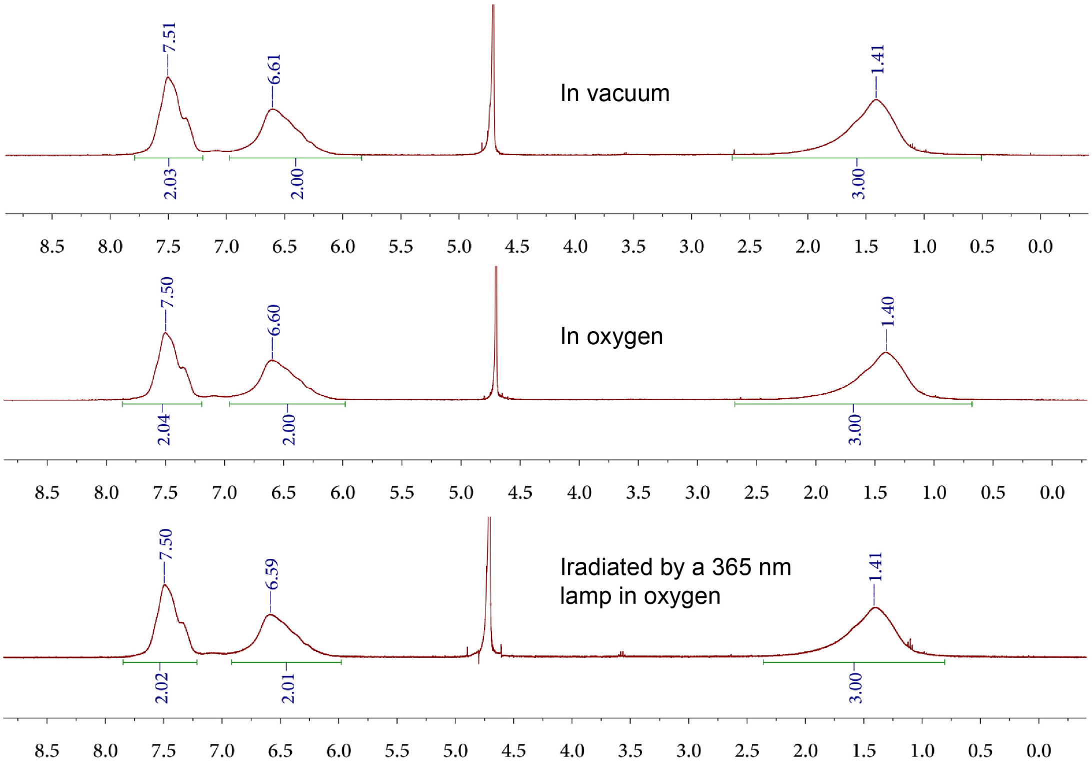


**Supplementary Figure 26.** $^{1}H$ NMR spectra of PSSNa polymer in $D_2O$ after placed in vacuum, exposed to oxygen for 3 hours or irradiated by a 365 nm lamp for 5 min in oxygen, respectively.

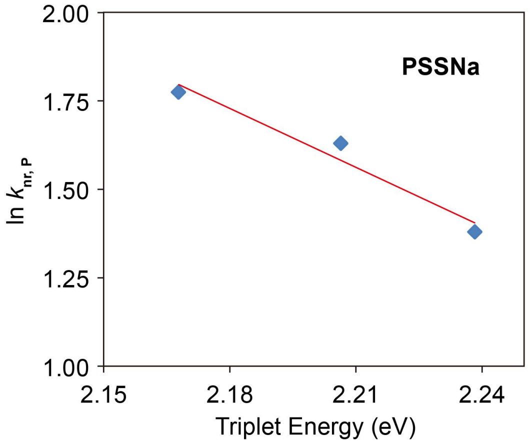


**Supplementary Figure 27.** The natural log of the non-radiative decay rate plotted against triplet energy for PSSNa polymer under different excitation wavelengths.

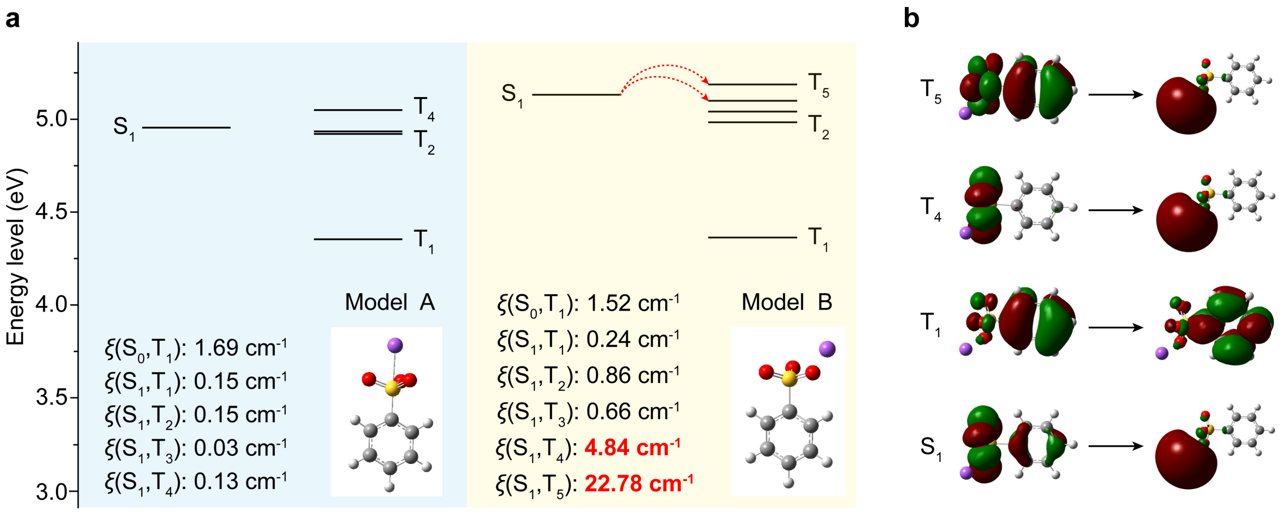


**Supplementary Figure 28. a,** Calculated energy diagram and spin-orbit coupling ($\xi$) for the chosen monomers, including model A and B. **b,** The Natural transition orbitals (NTOs) for the lowest single state ($S_1$) and the triplet states ($T_1$, $T_4$, $T_5$) of Model B.

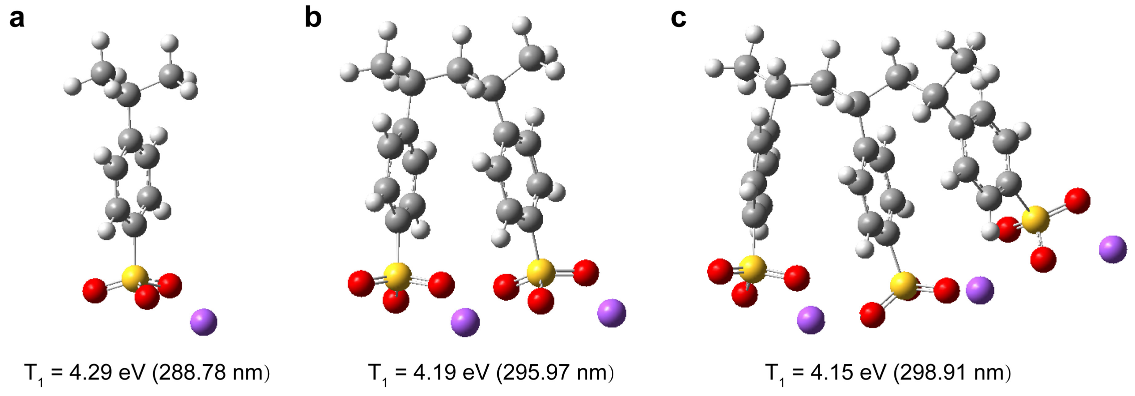


**Supplementary Figure 29. Calculated excitation energies for the lowest triplet state of selected segments in PSSNa polymers: monomer (a), dimer (b) and trimer (c).**

As shown in Supplementary Fig. 28, we calculated the energy levels and spin-orbit coupling constants of two models with ion at different positions. The large spin-orbit coupling constant ($S_1$ to $S_4$, $S_1$ to $S_5$) is ascribed to the lone electron pairs on the oxygen atoms and sulfur atom facilitating intersystem crossing, suggesting the model B is superior model for generating triplet exciton. Therefore, we build the dimer, trimer and hexamer based on model B with the ion near the sulfonic acid group (Supplementary Fig. 29). The energy of the lowest triplet state of PSSNa in dimer obviously decreased compared to the monomer, and for the trimer, it increased slightly. These results can also verify narrow change in phosphorescence emission at room temperature, arising from the aggregates of adjacent side groups in PSSNa. The blue-shifted phosphorescence emission at 77 K probably results from the remote side groups in PSSNa, and such constituent emit no phosphorescence at room temperature due to strong non-radiative transition.

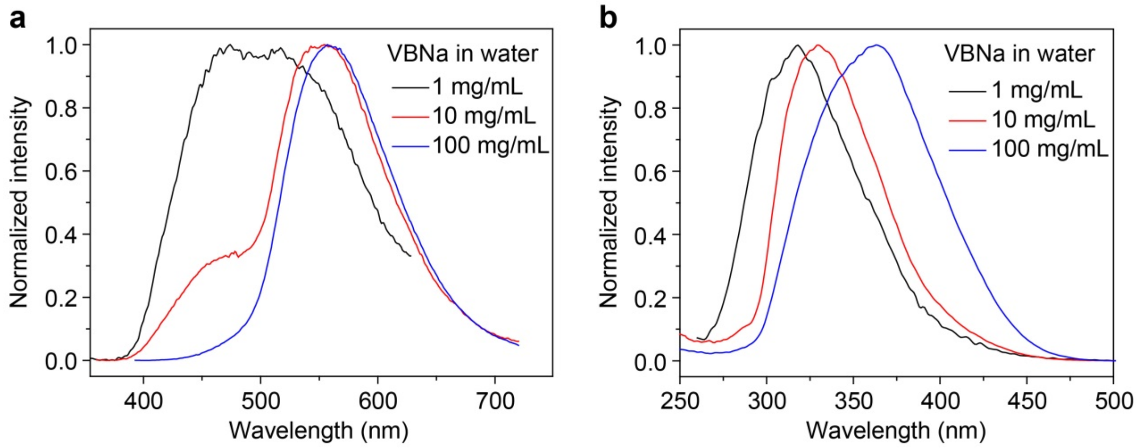


**Supplementary Figure 30. The photophysical properties of monomer (4-vinylbenzenesulfonic acid sodium) in deionized water at 77 K. a,** Phosphorescence spectra of monomer (4-vinylbenzenesulfonic acid sodium) in deionized water excited by optimal excitation wavelength. **b,** Excitation spectra of the monomer (4-vinylbenzenesulfonic acid sodium) in deionized water monitoring 560 nm at 77 K.

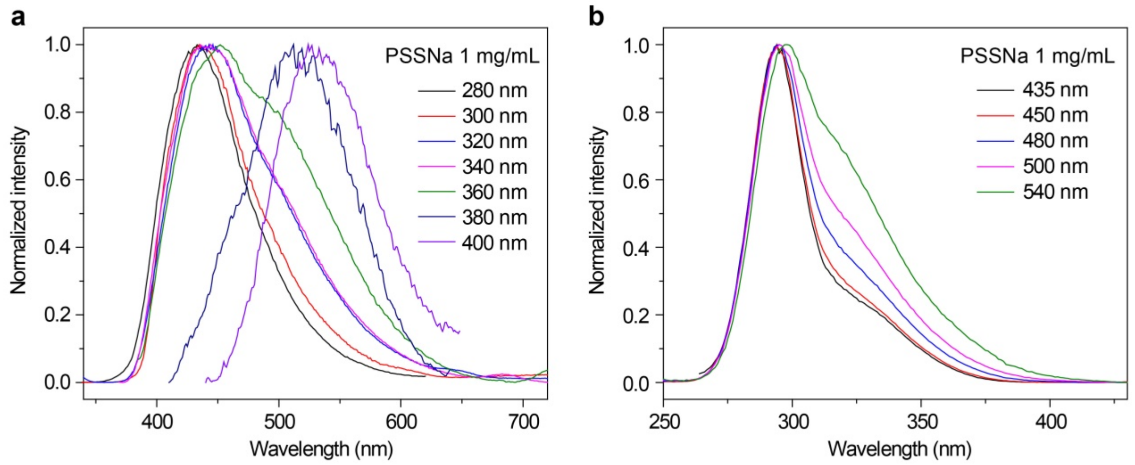


**Supplementary Figure 31. The photophysical properties of PSSNa in deionized water at 77 K. a**, Phosphorescence spectra of PSSNa (1 mg/mL) in deionized water excited by 280, 300, 320, 340, 360, 380 and 400 nm at 77 K. **b**, Excitation spectra of PSSNa (1 mg/mL) in deionized water monitoring 435, 450, 480, 500 and 540 nm at 77 K.

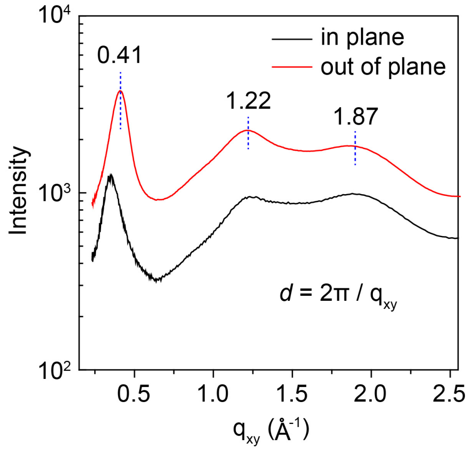


**Supplementary Figure 32.** 1D scattering profiles in the qz direction of 2D GI-WAXS pattern of dry PSSNa film.

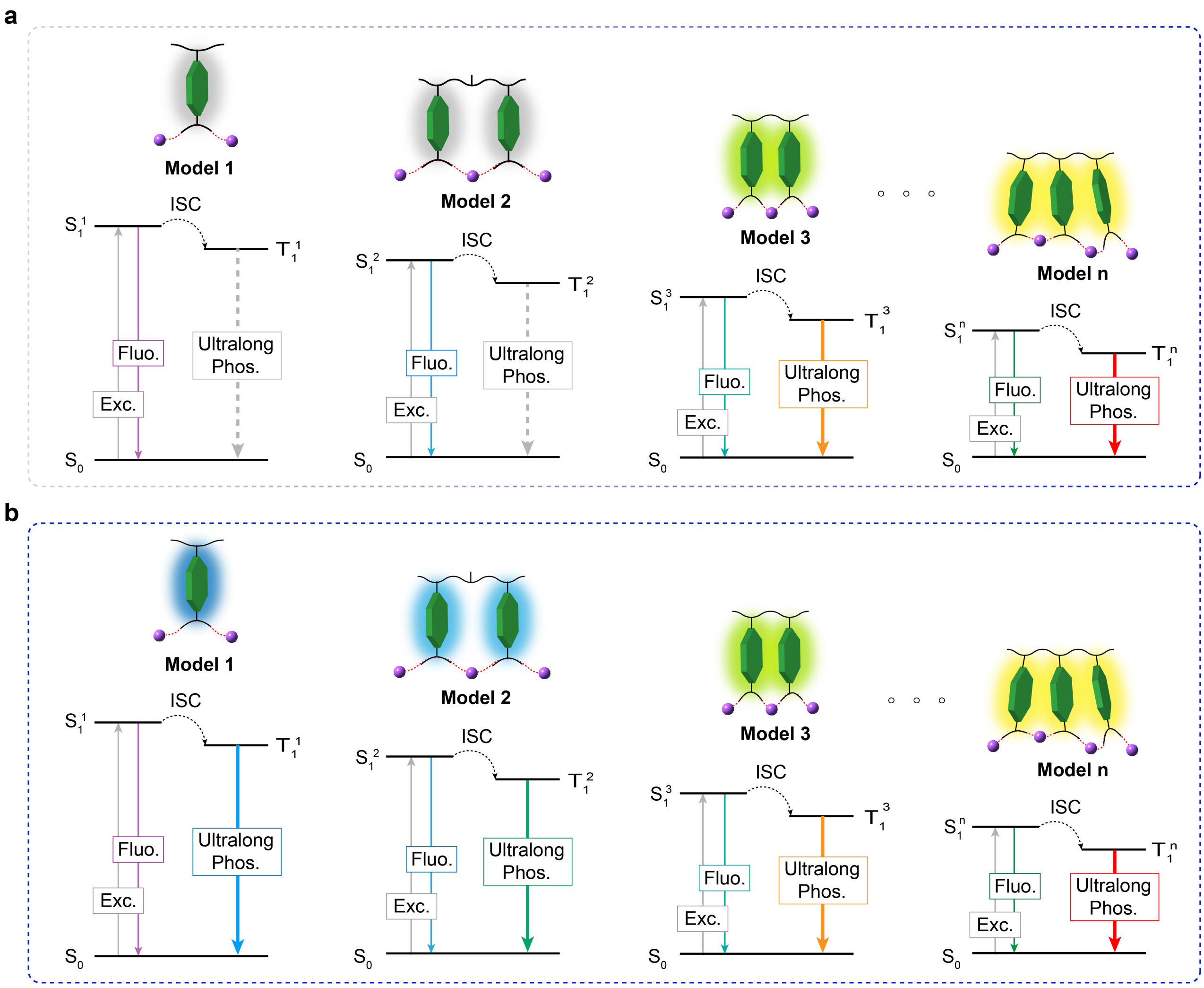


**Supplementary Figure 33. Proposed schematic energy diagrams of multicolor ultralong phosphorescence at room temperature (a) and at 77 K (b).** Note that different aggregates (Model 1, Model 2, Model 3, … Model n) in ionic polymers generate different singlet state and corresponding triplet state, thus contributing to multicolor phosphorescence emission from different aggregates of the chromophores.

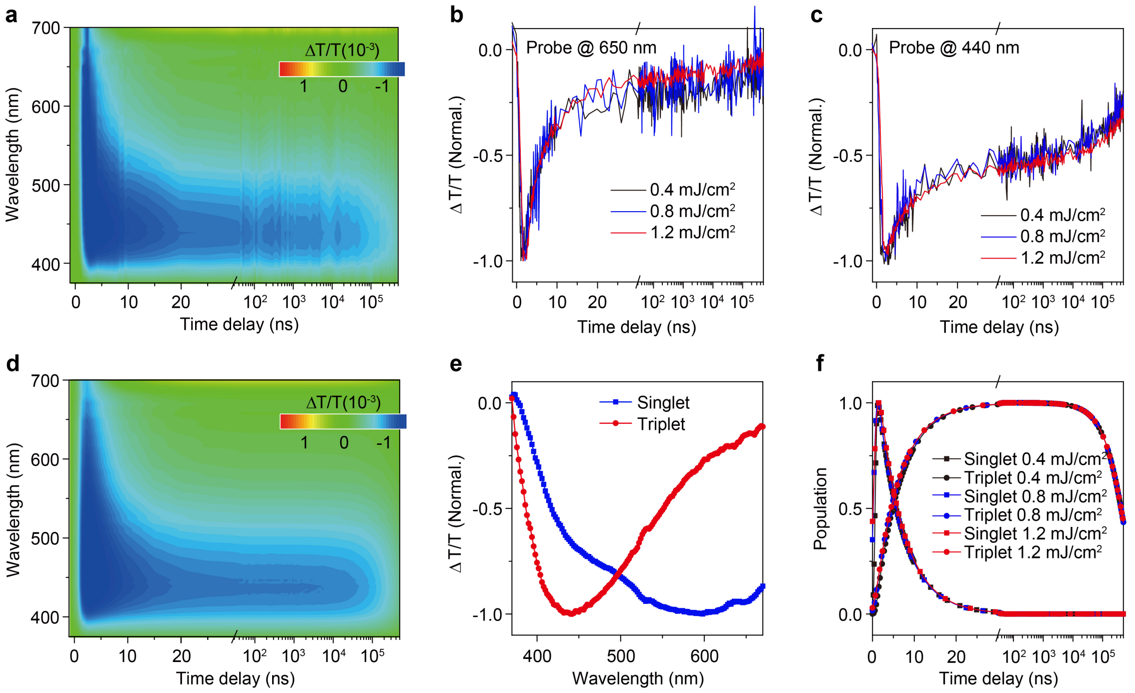


**Supplementary Figure 34. Excited state dynamics of PSSNa polymer. a,** Ns-resolved TA spectroscopic data of PSSNa polymer film recorded with a pump fluence of 1.2 mJ/cm2. Kinetic curves probed at **(b)** 650 nm and **(c)** 440 nm under pump at different fluences. **d,** Simulation TA data with the global fitting algorithm considering the conversion of ISC. **e,** TA spectral features of singlet and triplet states derived from the global fitting algorithm. **f,** The dynamic curves of singlet and triplet states obtained with TA data recorded at different pump fluences.

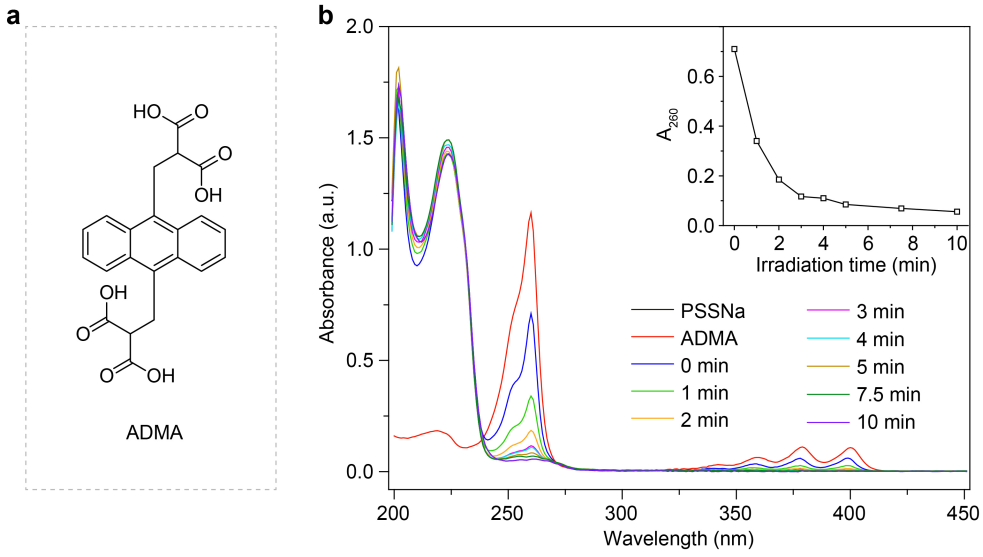


**Supplementary Figure 35. Detection of triplet-excited states in PSSNa polymer using ADMA. a**, Molecular structure of the ADMA molecules used for detecting the singlet oxygen species. **b**, Absorption spectra of ADMA under irradiation at 365 nm for different periods of time (0, 1, 2, 3, 4, 5, 7.5 and 10 min). Inset: plot of function relation of absorbance at 260 nm and irradiation time.

To further confirm the phosphorescence nature of the long-lived emission, an experiment on singlet oxygen detection was carried out. Notably, upon photoexcitation the energy transfer (ET) between the triplet state of the material and the ground state of molecular oxygen ($^3O_2$) can lead to the generation of an electronically excited state of molecular oxygen, i.e. singlet oxygen ($^1O_2$). Then, anthracene-9,10-diyl-bis-methylmalonate (ADMA) is utilized to detect the generated $^1O_2$ as a chemical trap by which this process can be quantitatively monitored using a UV-vis spectrophotometer. As a result, if there are triplet states and $^1O_2$ generated in the system upon photo-excitation, the characteristic absorption peaks of ADMA (260, 358, 378, and 399 nm) will be gradually decreased and eventually disappeared.[17,18] As shown in Supplementary Figure 35, the characteristic absorbance of ADMA gradually decreased with the increasing illumination time from 0 to 10 s, indicating the generation of the triplet-excited states upon photo-excitation of this purely organic compound.

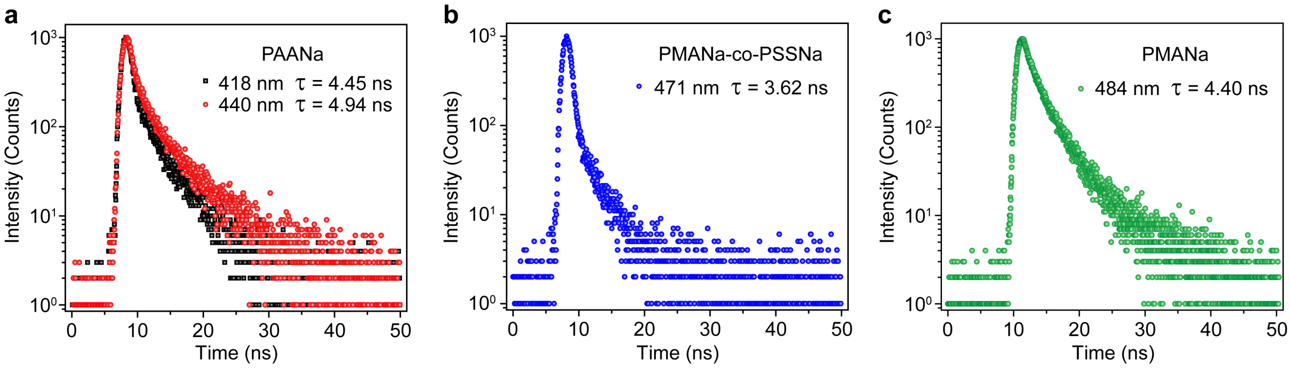


**Supplementary Figure 36.** Lifetime decay profiles of the fluorescence emission bands of (a) PAANa, (b) PMANa-co-PSSNa and (c) PMANa polymers under ambient conditions.

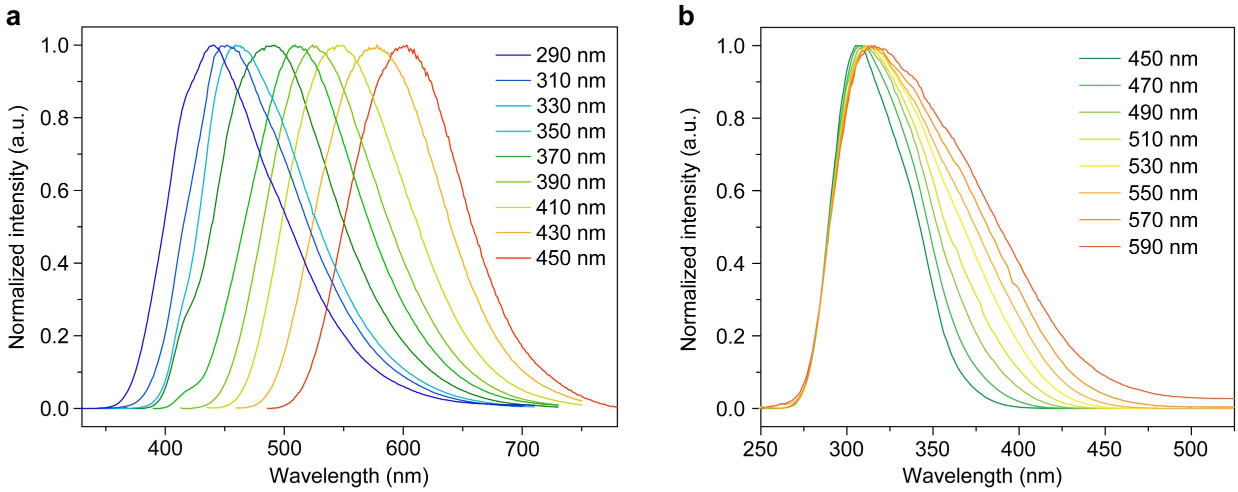


**Supplementary Figure 37. The phosphorescence properties of PAANa polymer at 77 K. a**, Excitation dependent phosphorescence spectra of PAANa polymer at 77 K. **b**, The excitation spectra of PAANa polymer monitoring 450, 470, 490, 510, 530, 550, 570 and 590 nm at 77 K.

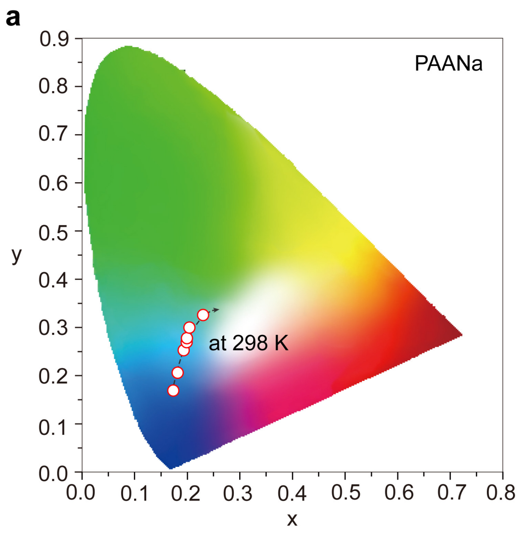


**Supplementary Figure 38. CIE chromaticity coordinate variation of phosphorescence emission color of PAANa along with excitation wavelength. a**, Red dots represent the CIE coordination of UOP for PAANa polymer at 298 K. **b**, Blue dots represent the CIE coordination of UOP for PAANa polymer at 77 K, respectively.

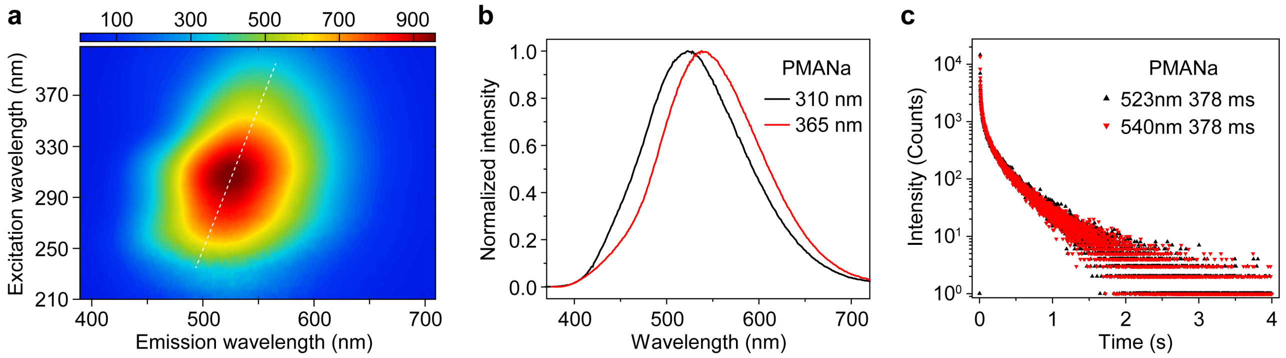


**Supplementary Figure 39. The photophysical properties of PMANa polymer in the solid state under ambient conditions. a**, Excitation-phosphorescence emission mapping of PMANa polymer. **b**, Phosphorescence spectra of PMANa polymer excited by 310 nm and 365 nm. **c**, The lifetime profiles of PMANa polymer monitoring 523 nm and 540 nm.

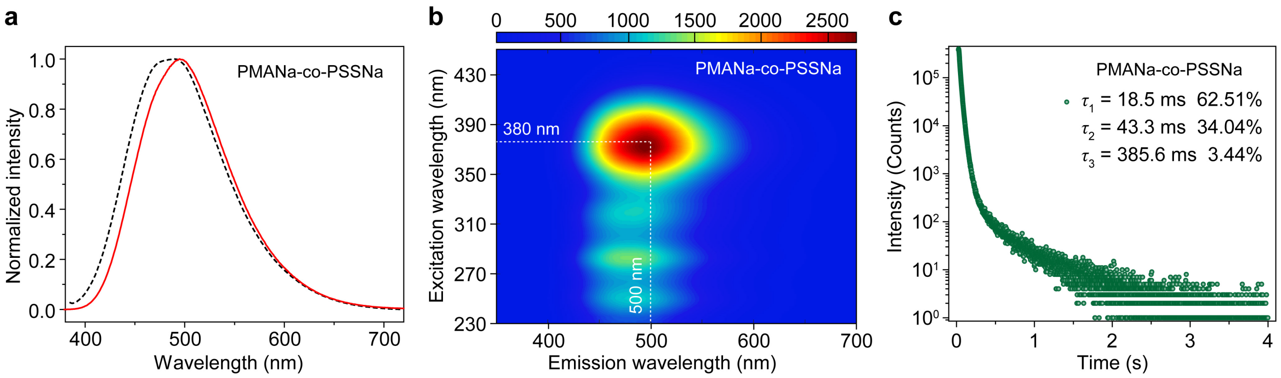


**Supplementary Figure 40. The photophysical properties of PMANa-co-PSSNa polymer in the solid state under ambient conditions. a**, Normalized photoluminescence (black, dash line) and phosphorescence (red, solid line) spectra of PMANa-co-PSSNa polymer excited at 365 nm. **b**, Excitation-phosphorescence emission mapping of PMANa-co-PSSNa polymer. **c**, The lifetime profiles of PMANa-co-PSSNa polymer monitoring 500 nm.

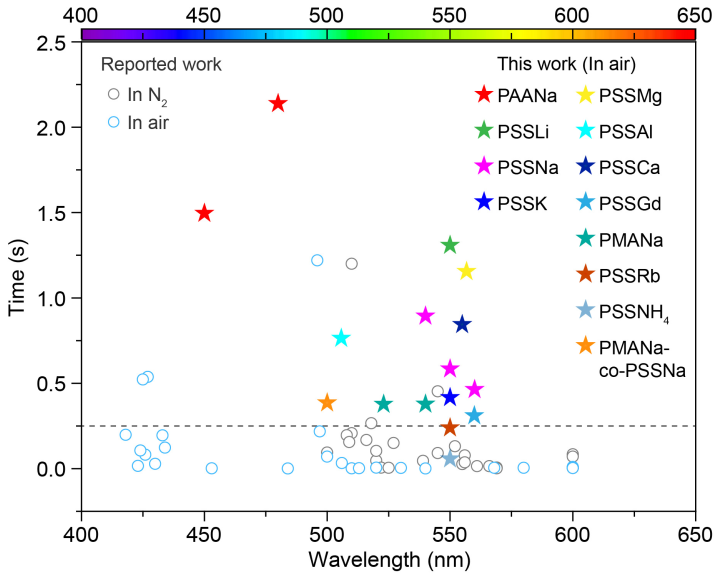


**Supplementary Figure 41. Distribution showing phosphorescence lifetime and emission peak of non-doped luminescence polymers under ambient conditions[1-16].**

**Supplementary Table 1. Photoluminescence quantum yields of PSSNa polymer at room temperature and 77 K.**

| Compounds | Excitation wavelength (nm) | Luminescence quantum yields (%) | |
|---|---|---|---|
| | | 298 K | 77 K |
| PSSNa | 330 | 4.89 | 10.47 |
| | 360 | 5.33 | 10.79 |
| | 400 | 6.84 | 12.24 |

**Supplementary Table 2. Fluorescence lifetimes ($\tau$) of polymers mentioned.**

| Compounds | Wavelength (nm) | Fluorescence | | | | |
|---|---|---|---|---|---|---|
| | | $\tau_1$ (ns) | $A_1$ (%) | $\tau_2$ (ns) | $A_2$ (%) | $\langle\tau\rangle$ (ns) |
| PSSNa | 397 | 1.46 | 73.58 | 4.13 | 26.42 | 2.17 |
| PSSLi | 397 | 1.38 | 63.97 | 4.53 | 36.03 | 2.51 |
| PSSK | 397 | 1.44 | 59.78 | 4.48 | 40.22 | 2.66 |
| PSSRb | 397 | 1.44 | 73.11 | 4.67 | 26.89 | 2.31 |
| $PSSNH_4$ | 397 | 1.31 | 63.01 | 3.76 | 36.99 | 2.22 |
| PSSMg | 397 | 1.56 | 63.38 | 4.22 | 36.62 | 2.53 |
| PSSAl | 420 | 1.46 | 65.10 | 3.19 | 34.90 | 2.06 |
| PSSGd | 423 | 0.53 | 59.62 | 2.39 | 40.38 | 1.28 |
| PSSCa | 411 | 1.31 | 60.83 | 4.26 | 39.17 | 2.47 |
| PAANa | 418 | 0.82 | 54.00 | 4.45 | 46.00 | 2.49 |
| PMANa | 484 | 1.56 | 48.03 | 4.40 | 51.97 | 3.03 |
| PMANa-co-PSSNa | 471 | 0.64 | 75.78 | 3.62 | 24.22 | 1.36 |
| PSS | 388 | 1.32 | 77.81 | 8.75 | 22.19 | 2.97 |

**Supplementary Table 3. Phosphorescence lifetimes ($\tau$) of PSSNa polymer in dry solid state and exposed to the air (humidity: 55%) for different time under ambient conditions.**

| Compounds | Wavelength (nm) | Phosphorescence | | | | | | |
|---|---|---|---|---|---|---|---|---|
| | | $\tau_1$ (ms) | $A_1$ (%) | $\tau_2$ (ms) | $A_2$ (%) | $\tau_3$ (ms) | $A_3$ (%) | $\langle\tau\rangle$ (ms) |
| Dry PSSNa Polymer | 540 | 42.50 | 17.42 | 212.68 | 41.68 | 894.50 | 40.90 | 461.9 |
| | 550 | 20.29 | 19.93 | 129.17 | 47.11 | 584.47 | 32.96 | 257.5 |
| | 560 | 19.45 | 20.14 | 95.42 | 46.53 | 463.54 | 33.32 | 202.8 |
| PSSNa 5 min | 550 | 17.95 | 26.89 | 98.45 | 45.05 | 472.92 | 28.05 | 181.8 |
| PSSNa 1 h | 550 | 4.60 | 56.44 | 112.00 | 43.56 | - | - | 51.4 |
| PSSNa 2 h | 550 | 1.04 | 34.77 | 3.97 | 43.96 | 24.05 | 21.27 | 7.2 |
| PSSNa 3 h | 550 | 0.90 | 23.57 | 4.55 | 42.69 | 20.57 | 33.75 | 9.1 |

**Supplementary Table 4. Phosphorescence lifetimes ($\tau$) of different ionic polymers in dry solid state under ambient conditions.**

| Compounds | Wavelength (nm) | Phosphorescence | | | | | | | | |
|---|---|---|---|---|---|---|---|---|---|---|
| | | $\tau_1$ (ms) | $A_1$ (%) | $\tau_2$ (ms) | $A_2$ (%) | $\tau_3$ (ms) | $A_3$ (%) | $\tau_4$ (ms) | $A_4$ (%) | <τ> (ms) |
| PSSLi | 550 | 32.42 | 14.87 | 116.50 | 38.27 | 381.34 | 35.53 | 1308.43 | 11.33 | 333.1 |
| PSSK | 550 | 21.17 | 33.72 | 89.94 | 49.21 | 416.56 | 17.07 | - | - | 122.5 |
| PSSRb | 550 | 19.79 | 32.43 | 65.48 | 52.13 | 239.51 | 15.44 | - | - | 77.5 |
| $PSSNH_4$ | 550 | 2.70 | 46.27 | 12.70 | 41.68 | 57.16 | 12.06 | - | - | 13.4 |
| PSSMg | 557 | 77.97 | 23.68 | 330.1 | 49.05 | 1152.2 | 27.28 | | | 494.7 |
| PSSAl | 506 | 87.52 | 22.65 | 269.8 | 65.99 | 765.3 | 11.36 | | | 284.8 |
| PSSGd | 560 | 27.42 | 30.58 | 99.74 | 53.15 | 314.9 | 16.27 | | | 112.6 |
| PSSCa | 555 | 57.80 | 31.81 | 218.1 | 52.94 | 845.3 | 15.25 | | | 262.8 |
| PAANa | 450 | 33.43 | 1.28 | 135.81 | 27.06 | 335.45 | 57.41 | 1496.54 | 14.26 | 443.2 |
| | 480 | 72.86 | 6.02 | 222.24 | 32.83 | 595.84 | 43.64 | 2139.05 | 17.51 | 711.9 |
| PMANa | 523 | 6.37 | 11.61 | 69.88 | 35.76 | 377.99 | 52.63 | - | - | 224.7 |
| | 540 | 5.58 | 12.92 | 65.91 | 37.71 | 378.28 | 49.36 | - | - | 212.3 |
| PMANa-co-PSSNa | 500 | 18.50 | 62.51 | 43.32 | 34.04 | 385.56 | 3.44 | - | - | 39.6 |
| PSS | 564 | 9.22 | 25.37 | 64.03 | 41.10 | 416.20 | 33.52 | - | - | 168.2 |

**Supplementary Table 5. Dynamic photophysical parameters of ultralong phosphorescence for ionic polymers.**

| Compounds | $\lambda_{ex}$ (nm) | $\Phi_F$ (%) | $\tau_F$ (ns) | $\Phi_P$ (%) | $\tau_P$ (ms) | $k_{r,F}$ [$s^{-1}$]$^{a)}$/$10^7$ | $k_{nr,F}$ [$s^{-1}$]$^{b)}$/$10^9$ | $k_{ISC}$ [$s^{-1}$]$^{c)}$/$10^7$ | $k_{r,P}$ [$s^{-1}$]$^{d)}$ | $k_{nr,P}$ [$s^{-1}$]$^{e)}$ | $T$ (K) |
|---|---|---|---|---|---|---|---|---|---|---|---|
| PSSNa | 554 | 4.11 | 4.13 | 0.79 | 249.8 | 1.00 | 0.230 | 0.190 | 0.031 | 3.972 | 298 |
| | 562 | 4.38 | 3.77 | 0.94 | 194.1 | 1.16 | 0.251 | 0.250 | 0.049 | 5.103 | 298 |
| | 572 | 4.45 | 4.81 | 2.40 | 165.5 | 0.92 | 0.194 | 0.499 | 0.145 | 5.897 | 298 |
| | 440 | 6.36 | 2.57 | 4.12 | 215.1 | 2.47 | 0.348 | 1.602 | 0.191 | 4.457 | 77 |
| | 516 | 3.22 | 4.3 | 7.57 | 465.0 | 0.75 | 0.207 | 1.760 | 0.163 | 1.988 | 77 |
| | 544 | 7.51 | 5.95 | 4.73 | 924.2 | 1.26 | 0.147 | 0.795 | 0.051 | 1.031 | 77 |
| PSSLi | 550 | 4.48 | 4.48 | 0.92 | 333.1 | 1.00 | 0.211 | 0.206 | 0.028 | 2.974 | 298 |
| PSSK | 550 | 4.26 | 4.53 | 1.54 | 122.5 | 0.94 | 0.208 | 0.340 | 0.126 | 8.038 | 298 |
| PSSRb | 550 | 3.36 | 3.76 | 0.44 | 77.5 | 0.89 | 0.256 | 0.117 | 0.057 | 12.846 | 298 |
| $PSSNH_4$ | 550 | 4.74 | 4.67 | 0.96 | 13.4 | 1.02 | 0.202 | 0.205 | 0.713 | 73.914 | 298 |
| PSSMg | 557 | 9.51 | 4.22 | 2.79 | 494.7 | 2.25 | 0.208 | 0.662 | 0.056 | 1.965 | 298 |
| PSSAl | 506 | 6.29 | 3.19 | 3.71 | 284.8 | 1.97 | 0.282 | 1.164 | 0.130 | 3.381 | 298 |
| PSSCa | 555 | 7.52 | 4.26 | 1.98 | 262.8 | 1.77 | 0.212 | 0.464 | 0.075 | 3.730 | 298 |
| PSSGd | 560 | 4.07 | 2.39 | 1.63 | 112.6 | 1.70 | 0.395 | 0.682 | 0.145 | 8.736 | 298 |
| PAANa | 450 | 3.81 | 4.45 | 5.79 | 443.2 | 0.86 | 0.203 | 1.301 | 0.131 | 2.126 | 298 |
| PMANa | 523 | 2.43 | 4.4 | 2.77 | 224.7 | 0.55 | 0.215 | 0.629 | 0.123 | 4.327 | 298 |
| PMANa-co-PSSNa | 500 | 0.92 | 3.62 | 13.08 | 39.6 | 0.26 | 0.238 | 3.612 | 3.302 | 21.951 | 298 |
| PSS | 564 | 4.46 | 8.75 | 0.14 | 168.2 | 0.51 | 0.109 | 0.016 | 0.008 | 5.937 | 298 |

a) $k_r^{Fluo} = \phi_{Fluo} / \tau_{Fluo}$; b) $k_{nr}^{Fluo} = (1-\phi_{Fluo}-\phi_{Phos}) / \tau_{Fluo}$; c) $k_{isc} = \phi_{Phos} / \tau_{Fluo}$; d) $k_r^{Phos} = \phi_{Phos} / \tau_{Phos}$; e) $k_{nr}^{Phos} = (1-\phi_{Phos}) / \tau_{Phos}$

**Reference**


1. Zhang, G., Palmer, G. M., Dewhirst, M. W. & Fraser, C. L. A dual-emissive-materials design concept enables tumour hypoxia imaging. *Nat. Mater.* **8**, 747-751 (2009).
2. Ogoshi, T. et al. Ultralong room-temperature phosphorescence from amorphous polymer poly(styrene sulfonic acid) in air in the dry solid state. *Adv. Funct. Mater.* **28**, 1707369-1707376 (2018).
3. Zhou, Q. *et al.* Clustering-triggered emission of nonconjugated polyacrylonitrile. *Small.* **12**, 6586-6592 (2016).
4. Kwon, M. S. *et al.* Suppressing molecular motions for enhanced room-temperature phosphorescence of metal-free organic materials. *Nat. Commun.* **6**, 8947-8956 (2015).
5. DeRosa, C. A. *et al.* Tailoring oxygen sensitivity with halide substitution in difluoroboron dibenzoylmethane polylactide materials. *ACS. Appl. Mater. Interfaces.* **7**, 23633-23643 (2015).
6. Zhang, G., Evans, R. E., Campbell, K. A. & Fraser, C. L. Role of boron in the polymer chemistry and photophysical properties of difluoroboron-dibenzoylmethane polylactide. *Macromolecules* **42**, 8627-8633 (2009).

7. Zhang, G., Fiore, G. L., St. Clair, T. L. & Fraser, C. L. Difluoroboron dibenzoylmethane PCL-PLA block copolymers:matrix effects on room temperature phosphorescence. *Macromolecules* **42**, 3162-3169 (2009).
8. Samonina-Kosicka, J., DeRosa, C. A., Morris, W. A., Fan, Z. C. & Fraser, L. Dual-emissive difluoroboron naphthyl-phenyl beta-diketonate polylactide materials: effects of heavy atom placement and polymer molecular weight. *Macromolecules* **47**, 3736-3746 (2014).
9. DeRosa, C. A. *et al.* Oxygen sensing difluoroboron dinaphthoylmethane polylactide. *Macromolecules* **48**, 2967-2977 (2015).
10. Chen, X. *et al.* Versatile room-temperature-phosphorescent materials prepared from N-substituted naphthalimides:emission enhancement and chemical conjugation. *Angew. Chem. Int. Ed.* **55**, 9872-9876 (2016).
11. DeRosa, C. A. *et al.* Oxygen sensing difluoroboron $\beta$-diketonate polylactide materials with tunable dynamic ranges for wound imaging. *ACS Sensors* **1**, 1366-1373 (2016).
12. Kerr, C. *et al.* Luminescent difluoroboron beta-diketonate PLA-PEG nanoparticle. *Biomacromolecules* **18**, 551-561 (2017).
13. Wang, T. *et al.* Dual-emissive waterborne polyurethanes prepared from naphthalimide derivative. *Polymers* **9**, 411-420 (2017).
14. Sun, W. *et al.* Protonation-induced room-temperature phosphorescence in fluorescent polyurethane. *J. Phys. Chem. A*. **121**, 4225-4232 (2017).
15. Kanosue, K. & Ando, S. Polyimides with heavy halogens exhibiting room-temperature phosphorescence with very large stokes shifts. *ACS Macro Letters* **5**, 1301-1305 (2016).
16. Chen, H., Yao, X., Ma, X. & Tian, H. Amorphous, efficient, room-temperature phosphorescent metal-free polymers and their applications as encryption Ink. *Adv. Optical Mater.* **4**, 1397-1401 (2016).
17. Ma, J., Chen, J. Y., Idowu, M. & Nyokong, T. Generation of singlet oxygen via the composites of water-soluble thiol-capped CdTe quantum dots-sulfonated aluminum phthalocyanines. *J. Phys. Chem. B* **112,** 4465-4469 (2008).
18. Çamur, M., Durmus, M., Bulut, M. Highly singlet oxygen generative water-soluble coumarin substituted zinc(II) phthalocyanine photosensitizers for photodynamic therapy. *Polyhedron* **41**, 92-103 (2012).
