# Supplementary material for: Enabling long-lived organic room temperature phosphorescence in polymers by subunit interlocking
Source: Nat Commun. 2019 Sep 18;10:4247. doi: 10.1038/s41467-019-11749-x (PMC6751207; doi:10.1038/s41467-019-11749-x)
Supplement: Supplementary file 2 — Description of Additional Supplementary Files [file 41467_2019_11749_MOESM2_ESM.pdf]

Description of Additional Supplementary Files

File Name: Supplementary Movie 1
Description: PSSNa polymer in the solid state was light-yellow under room light. When excited by a 365 nm UV lamp, PSSNa polymer presented blue. After the removal of the excitation light source, the emission color turned yellow. And yellow ultralong emission can be observed by the naked eyes for several seconds under ambient conditions.

File Name: Supplementary Movie 2
Description: PSSLi polymer in the solid state was transparent films under room light. PSSNa polymer exhibited blue under a 365 nm lamp on. After ceasing the irradiation, the emission color turned yellow-green. Surprise, yellow ultralong phosphorescence can last for more than fifteen seconds under ambient conditions.
